# Molar occlusion and jaw roll in early crown mammals

**DOI:** 10.1038/s41598-020-79159-4

**Published:** 2020-12-24

**Authors:** Kai R. K. Jäger, Richard L. Cifelli, Thomas Martin

**Affiliations:** 1 Section Palaeontology, Institute of Geosciences, Rheinische Friedrich-Wilhelms-Universität Bonn, Nussallee 8, 53115 Bonn, Germany; 2 Oklahoma Museum of Natural History, 2401 Chautauqua Ave, Norman, OK 73072 USA

**Keywords:** Evolution, Palaeontology, Biomechanics

## Abstract

Triconodontidae are considered the first carnivorous crown mammals. A virtual reconstruction of the masticatory cycle in the Late Jurassic *Priacodon* showed that triconodontid dental function is characterized by precise cutting on elongated crests. The combination of traits linked to both carnivorous diets (e.g. fore-aft cutting edges) and insectivorous diets (transverse crests and lobes) suggests a varied faunivorous diet appropriate to the small body size of most triconodontids. Total length of molar shear decreased with wear, suggesting a dietary shift during ontogeny. Embrasure occlusion is confirmed for *P. fruitaensis* as indicated by premolar positioning, facet orientation, and collision areas. Embrasure occlusion is considered a general feature of all Eutriconodonta, whereas the previously assumed *Morganucodon*-like pattern is limited to few early mammaliaforms. Unlike modern carnivores, significant roll of around 10° of the active hemimandible occurred during the power stroke. Roll was likely passive in Triconodontidae in contrast to active roll described for extant therians. The triconodontid molar series was highly uniform and adapted to a precise fit, with self-sharpening lower molar cusps. Whereas the uniformity ensured good cutting capabilities, it likely put the dentition under greater constraints, conserving the highly stereotyped nature of triconodontid molars for 60–85 Ma.

## Introduction

Triconodontids are a clade of the eutriconodontans^1,2^, which is a clade of early crown mammals with a fossil record from the Late Jurassic through the Late Cretaceous^3–8^. It has been well established that stem mammaliaforms with triconodont-like molars, such as *Morganucodon*, are phylogenetically distinct and separated from the eutriconodontans^9^ by many derived features of the latter groups, including tooth wear characteristics^10^. Triconodontidae differ from stem mammaliaforms with “triconodont-like” molar pattern, such as *Morganucodon*^9,10^, by the uniform shape and size of their cusps.

Triconodontidae are long-viewed as small but highly efficient carnivores^11,12^ with their molar row essentially forming a single battery of an almost identical cusp-valley sequence with a continuous cutting edge^12^. In more derived triconodontids, the accessory D/d cusp can be enlarged and integrated into that series (e.g. *Alticonodon*)^3,13^. Most taxa exhibit an interlocking system between adjacent molars, with cusp d fitting in an embayment anterior to cusp b of the succeeding molar^3,4,14–19^.

In this study the occlusion, jaw movement and jaw morphology of the three earliest occurring taxa of Triconodontidae *Priacodon*, *Triconodon*, and *Trioracodon* were examined^2,6^.

**Figure 1:**
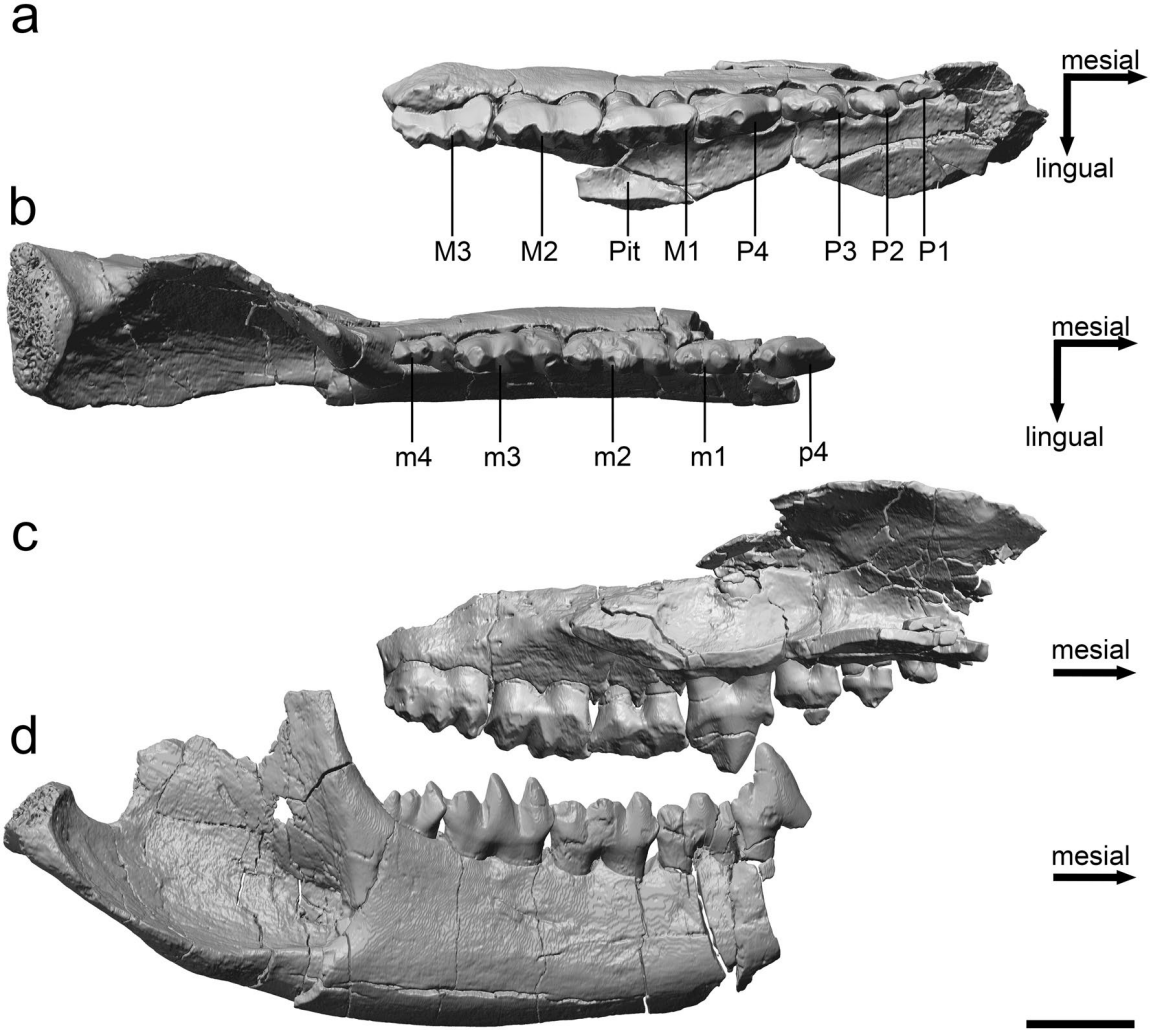
Right dentition of *Priacodon fruitaensis* (LACM 120451). (**a**) Upper tooth row in occlusal view; (**b**) lower tooth row in occlusal view; (**c**) upper tooth row in lingual view (mirrored for better comparison); (**d**) lower tooth row in buccal view. Note that parts of the palatine have been digitally removed for better visibility and that p4 was repositioned in the virtual model to its natural position because it was damaged and shifted upwards during fossilization. The posterior part of the jaw, beginning with the ascending ramus, was shifted laterally due to damage and the masseteric fossa, therefore, appears larger in occlusal view. Scale bar equals 2.5 mm.

Molar morphology of these early Triconodontidae is so similar that the number of postcanine teeth has to be considered for distinction of the genera^4^. While their uniform tooth morphology causes problems for systematics, this makes it convenient to study occlusion and dental function of exemplary taxa, for the entire family. In this study micro-computed tomography (µCT) and 3D models were applied to re-examine and functionally analyse the dentition of the right ramus and maxilla of *P*. *fruitaensis*. The holotype, LACM 120451, is the only specimen of an early triconodontid with well-preserved, ipsilateral upper and lower dentitions (Fig. 1). The aim is to better understand the function and occlusion of Triconodontidae in general and to test existing hypotheses on the occlusal mode^20,21^, jaw roll^22^, and diet^11,12^. Understanding the dental function of Triconodontidae is of particular interest, as they represent early crown Mammalia with a modified version of the plesiomorphic triconodont tooth pattern, characterized by a high degree of uniformity and with little change from the Late Jurassic till the Late Cretaceous.

### Previous occlusal hypotheses

Simpson^20^ briefly discussed the occlusion of early Triconodontidae and summarized his reconstruction in an illustration depicting the relative positioning of upper and lower molars. This occlusal pattern was later also described for the early mammaliaform *Megazostrodon* and termed “embrasure occlusion”^21,23^. In this occlusal model, the main cusps (A/a) occlude between the antagonistic molars. Mills^21^ subsequently proposed a new model with cusp a of the lower molars of *Triconodon* and *Trioracodon* occluding between the upper cusps B and A, and cusp A occluding between cusps a and c, similar to *Morganucodon*^21^. This model, here referred to as one-on-one occlusion, argued that the tooth positioning proposed by Simpson^20^ would result in a mismatch of the ultimate premolars. However, this mismatch was inferred from upper and lower dentitions of different individuals. When Mills’^21^ proposed his hypothesis, only one specimen of Triconodontidae (*Trioracodon ferox* NHMUK PV OR 47781) with preserved matching second lower and upper molars was known. Because in this specimen m2 is only exposed in lingual aspect, it is of limited value for the interpretation of the occlusal relationships. In addition, Mills’ hypothesis was likely influenced by the occlusion of *Morganucodon*, which was analyzed in the same study^21^. In *Morganucodon* cusp a often occludes between cusps B and A, and cusp A always occludes between cusps a and c^10^. When Mills^21^ discussed the occlusion of *Morganucodon* and early Triconodontidae, both were considered to belong to “Triconodonta”^11^, which now are recognized to be paraphyletic, with Triconodontidae placed among crown mammals and *Morganucodon* as an early mammaliaform^1,4,9,24^. Although not specifically stated by Mills^21^, it is likely that his hypothesis on the occlusion of *Triconodon* and *Trioracodon* was influenced by the assumed close phylogenetic relationship to *Morganucodon*.

## Results and discussion

### OFA and occlusion

**Figure 2:**
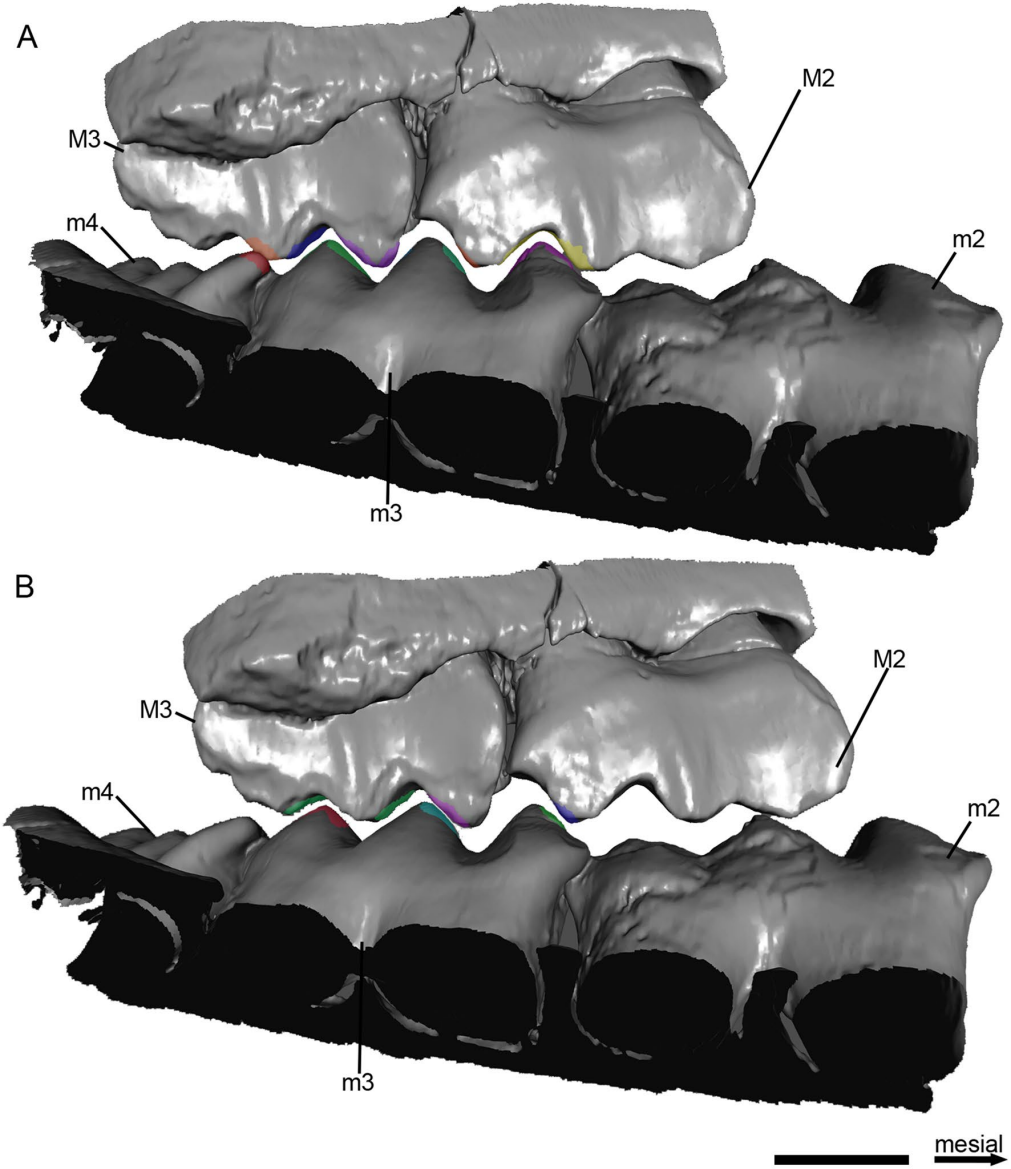
Alternative occlusal models of *P*. *fruitaensis* (right dentition, ventral lateral view, in the middle of power stroke rendered by simulation analyses of OFA). (**A**) after Simpson^20^ and (**B**), after Mills^21^. Although the cusp-valley system is homogenous minor differences in size of the individual cusps and valleys occur. These differences support the occlusion after Simpson^20^ since every cusp opposes a valley that matches its dimension. Colored areas represent the contact of the 3D models at this time stage of the analysis. Scale bar equals 1 mm.

In the OFA analysis (see Material and methods), the embrasure occlusion model^20^ performed better than the one-on-one occlusal model^21^ in several aspects. When the one-to-one occlusal model was applied to the dentition of *P. fruitaensis*, the ultimate premolars collided with each other in starting position of the power stroke. In contrast, embrasure occlusion resulted in a much better fit of the ultimate lower premolars (Supplementary Fig. 1) which contradicts Mills’^21^ argument on the premolar positioning. Further evidence is provided by the wear pattern on the ultimate lower molar (Supplementary Figs. 1 and 2D). Cusp a of m4 has a well-developed wear facet on its distobuccal side. Following Mills’^21^ occlusal mode, no antagonist could contact this facet (Fig. 2 and Supplementary Fig. 1). Embrasure occlusion, on the other hand, allows cusp A of M3 to contact this part of the small m4. This is further supported by the circular shape of the facet on cusp A of M3, which suggests that the larger cusp A cusp circumvented small cusp a of m4.

**Figure 3:**
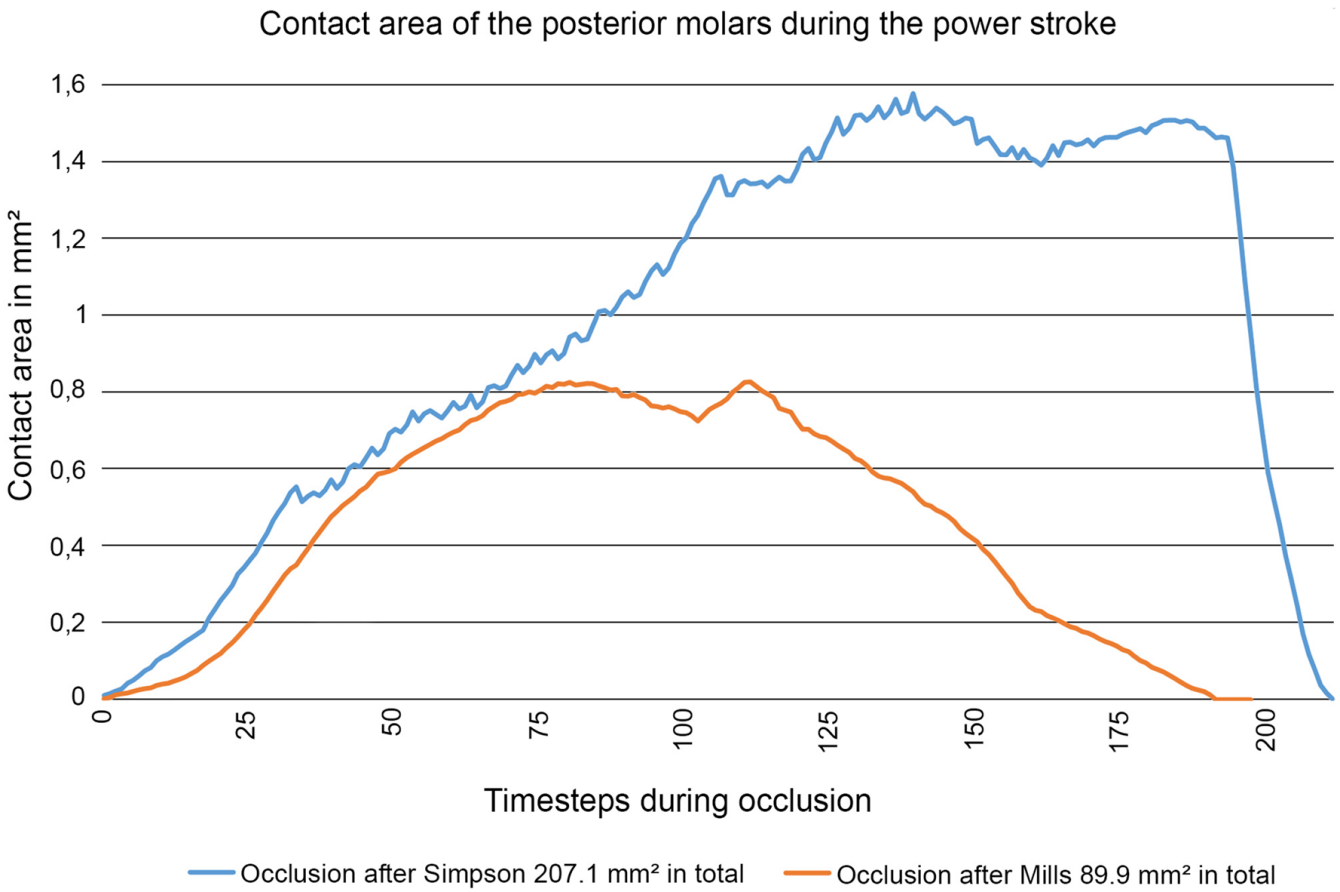
Quantitative comparison of the Simpson Model^20^ and the Mills Model^21^ for upper-lower molar occlusion, by OFA analysis of contact surfaces of *P*. *fruitaensis*. Y-axis: contact surface area of M2–M3 with m2–m4 (in mm^2^) converted from the collision area detected by the OFA software. X-axis: time steps (1–211) of the occlusal movement of a single power stroke. Simpson’s embrasure occlusion (blue) can achieve larger total contact area and longer duration than Mills’ *Morganucodon*-like occlusion. While more contact does not validate the hypothesis by itself, it supports the interpretation of a better fit for the Simpson model.

The OFA provides a quantitative assessment to compare and evaluate existing hypotheses on occlusion as has been demonstrated earlier for *Docodon*^25^. In the case of *P. fruitaensis*, embrasure occlusion results in larger contact area over a longer duration of the occlusal action (Fig. 3). While an increased occlusal contact is only one feature, and may not sufficient to fully validate an occlusal hypothesis, the better fit of the overall occlusion certainly is the strongest validation of this occlusal hypothesis.

At the beginning of the power stroke, both hypotheses result in a similar amount of contacts. While the cusps of the lower molars progress more deeply in the spaces between the cusps of the uppers, the difference in fit between the two occlusal hypotheses becomes apparent (Figs. 2 and 3). With the better fit of embrasure occlusion, the cusps of the lower molars enter more deeply into the valleys of the upper molars, resulting in more extensive contact (Fig. 3). Subsequently, the teeth can pass each other smoothly and therefore stay in contact over a greater part of the occlusal cycle. Under one-on-one occlusion, the cusps enter the valleys to a much lesser degree and the teeth either get stuck or, to finish the path, need to disengage before all cusps have fully slid past one another.

Mills’^21^ hypothesis of a *Morganucodon*-like occlusion in Triconodontidae was widely accepted in following decades^4,26^ and, in at least one case, a *Morganucodon*-type occlusal pattern has been ascribed to a Cretaceous (alticonodontine) triconodontid^17^.

With an embrasure occlusion confirmed for *P. fruitaensis*, we propose that this embrasure occlusion is a diagnostic feature in all of the Triconodontidae, given the overall similar molar morphology within the family. Accordingly, this pattern would be universal for known Eutriconodonta, as Gobiconodontidae have embrasure occlusion as well^4,27^.

This suggests that one-on-one occlusion was limited to *Morganucodon* and potentially a few other early mammaliaforms (e.g. *Dinnetherium*)^22^. When molar morphology is compared among all taxa with triconodont (cusp-in-line) dentitions, a notable difference is that cusps B/b of *Morganucodon* are relatively small compared to the main cusps A/a. This might have been necessary for the transition from embrasure occlusion toward the one-to-one occlusion of *Morganucodon*, and could explain why this type of occlusion did not evolve within the Triconodontidae, which have cusps of more uniform size and height.

Occlusion in tribosphenic mammals is characterized by a power stroke with two phases (I and II), separated by a directional change, that causes different orientations in striations on the wear facets^28^. Since all striations in *P*. *fruitaensis* follow the same orientation and the molar morphology leaves little room for directional changes, it can be concluded that the power stroke was single phased (phase I), which is common for pretribosphenic dentitions^29,30^.

At the beginning of the power stroke, most of the cusps come into contact in rapid succession (Supplementary Fig. 3). In the OFA analysis, this is demonstrated by an initial sharp increase in contact area at the beginning of the power stroke (Fig. 3). This is in marked contrast to “triconodont” dentitions with large A/a cusps (e.g. *Morganucodon*), where substantial contact occurs on these principal cusps well before on the smaller cusps^10^. The similar height of triconodontid molar cusps, together with their rapid occlusal contact, implies that precise alignment of the lower jaw, prior to the first contact, was required.

Simpson^12,31^ postulated that during the initial orthal movement, the lower molar crests passed along the upper molar crests. Subsequently, during the later stage of the power stroke, when the lower molars moved lingually and distally, their crests passed along the ridge-like cusps of the uppers. This hypothesis is confirmed based on the OFA. The power stroke is oriented straight orthally, with a slight distal (backward) deviation, due to the orientation of the valleys in the upper molars. Only vertical and transverse jaw movements were proposed^12,31^. However, a third, rotational movement—roll of the active hemimandible around its longitudinal axis—was present and resulted in wear facets with different orientation during the latter part of the power stroke.

### Jaw roll

Roll^32^ during the chewing cycle of both the working-side (WS) or active hemimandible and the balancing-side (BS) or inactive hemimandible has been observed in several extant mammals^33–36^ and was also inferred for Mesozoic mammals and mammaliaforms^32,36,37^. Roll requires a mobile symphysis and was hypothesized to have been essential for the evolution of precise occlusion^36^. Roll was described for an unnamed triconodontid from the Cloverly Formation based on more vertical wear facets on the lower molars and more horizontal on the uppers^22^. This difference was explained with the necessity of a medial rotation of the lower jaw during occlusion^22^. Similarly-oriented facets are present in *Priacodon fruitaensis* specimen LACM 120451 (supplementary information). For the Cloverly triconodontid^22^, a roll of approximately 20° was illustrated in a schematic drawing of the working-side (active) hemimandible during power stroke. The OFA of LACM 120451 confirms the hypothesis that roll was present in the masticatory cycle of *Priacodon*. A comparison of paths with and without roll showed that no roll is required to produce the wear on the upper molars, because most of it is caused by the tips of the lower molar cusps entering the valleys between the upper molar cusps. However, the facets on the lower molars that extend far to the bases between the cusp valleys, as seen in m3 (Supplementary Figs. 2 and 4), require roll in order to make contact with the upper molars. A notable difference from the previous hypothesis^22^, however, is the amount of roll required (Supplementary Fig. 3 mesial view). Roll of approximately 10° of the active hemimandible during the power stroke is sufficient to create the observed wear facets. The degree of rotation proposed here is different from the previously suggested 20° of roll^21^. This can be explained by the position of the upper molars in the maxilla and the lower molars in the ramus (Supplementary Fig. 5). The former reconstruction^22^ assumed that the molars were positioned vertically, with their cusps pointing ventrally and dorsally, respectively. When the maxilla and jaw are virtually aligned (based on a horizontal position of the palatine and the transverse axis of the condyle, as well as a straight orientation of the premolars) and put into occlusion, it becomes evident that the upper molars in the maxilla are inclined lingually and the lower molars are slightly inclined buccally. A similar inclination has been described for some Gobiconodontidae^2,27^ and *Morganucodon watsoni*^10^. While this inclination reduces the amount of roll required to create matching occlusion, a roll of ~ 10° of the active hemimandible during the power stroke is still higher than the average observed roll for the extant marsupial *Monodelphis*, where the active hemimandible during the power stroke was inclined mostly around 5° and in one case up to 10°^36^. In Triconodontidae, roll of the active hemimandible during the power stroke was limited to a medial inclination of the jaw.

**Figure 4:**
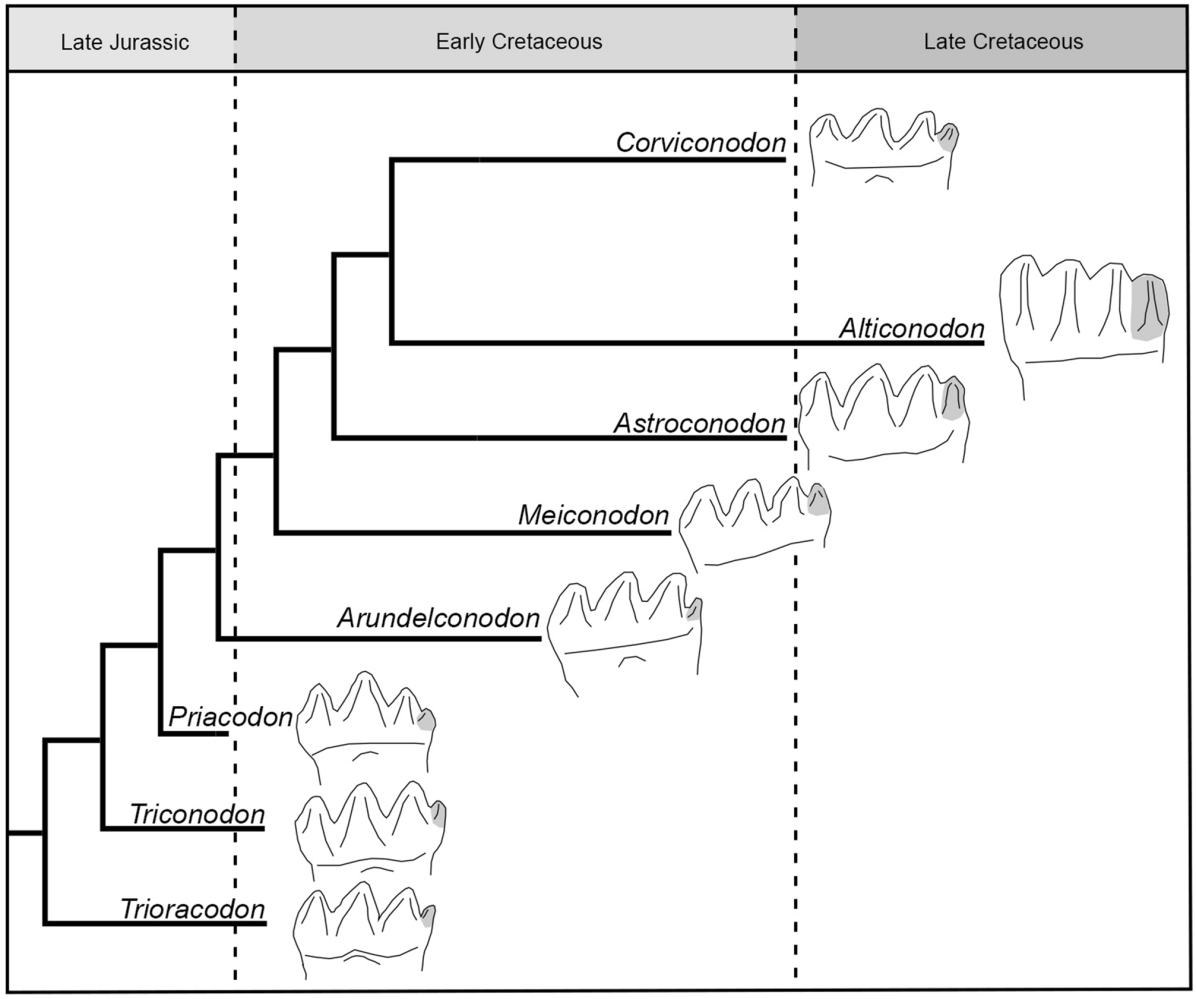
Evolutionary pattern of molar cusp size and proportion in Triconodontidae. Cusp size of the three main cusps becomes more similar, while cusp d increases in size. Its function changes from merely interlocking with the next posterior molar to an active unit during food processing. In this role it is initially still closely positioned next to cusp c (e.g. *Meiconodon*, *Astroconodon*). In the last lower molar of *Alticonodon,* it is a fully separated cusp that functions as a single unit in the tooth battery. *Astroconodon* and *Alticonodon* also show an increase in crown height. Tooth position varies, which can influence the relative size of the cusps to a small degree. The cusp dimensions of *Corviconodon* are approximations of the dimensions of unworn teeth based on the worn holotype of *C*. *utahensis*. *Priacodon* is based on the holotype of *P*. *ferox* since no tooth is complete in the holotype of *P*. *fruitaensis*. The former has greater differences in cusp size than the latter. The phylogeny is based on Martin et al.^2^.

**Figure 5:**
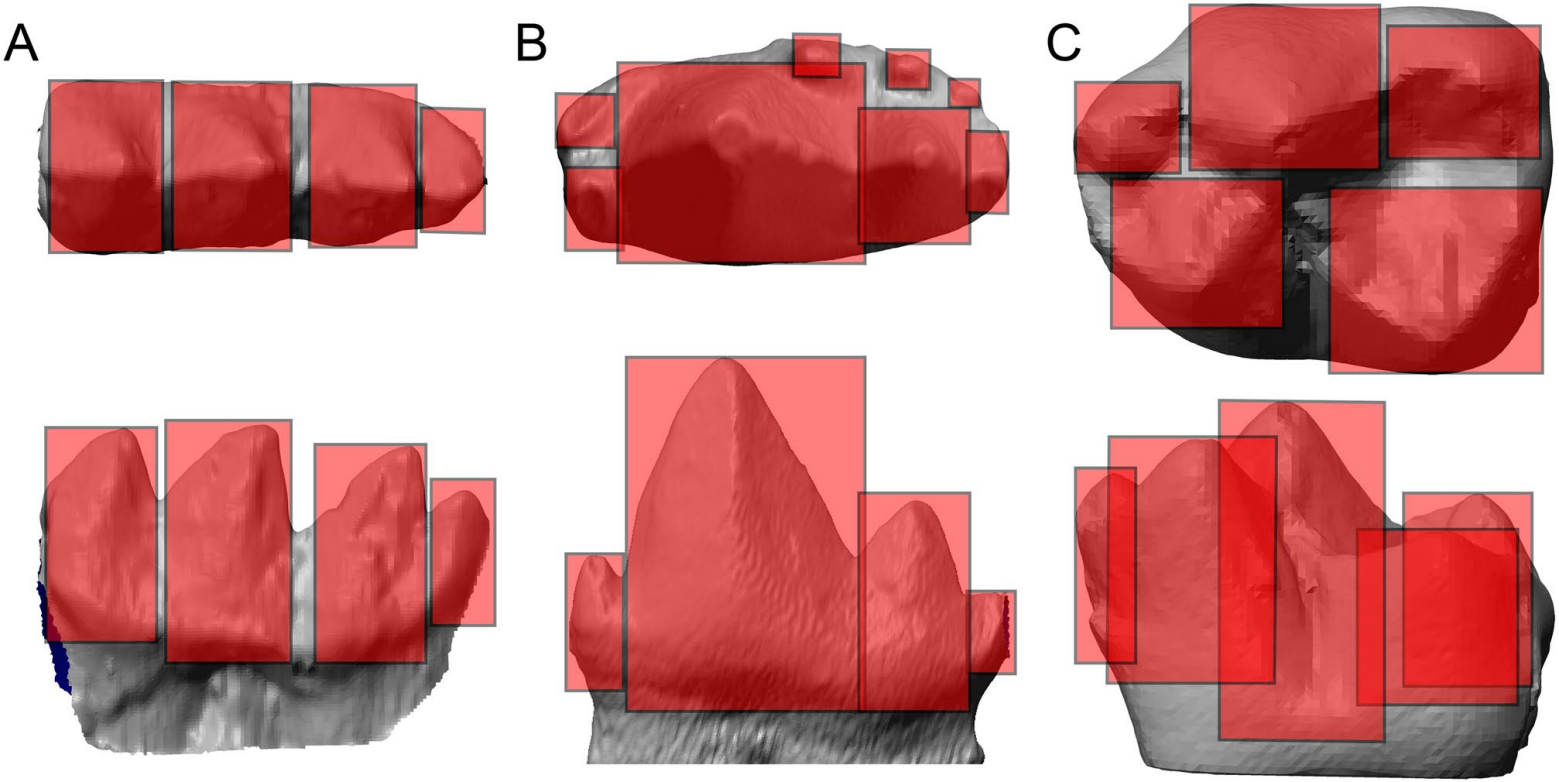
Comparative patterns of molar cusp size and height of Triconodontidae, relative to mammaliaforms and other mammals. (**A**) Derived triconodontid; (**B**) plesiomorphic triconodont molar of *Morganucodon* (UMZC Eo.CR1); and C, a tribosphenic molar of *Cantius ralstoni* (USGS 13634) in occlusal and lingual view. Squares are plotted over individual cusps to highlight them as functional units. It is apparent that the plesiomorphic triconodont molar and the tribosphenic molar are more heterogeneous in their cusp dimensions, while the triconodontid molar is more uniform. In combination with its precise and enclosed occlusal pattern, this uniformity could have placed constrains on the evolutionary development of the molar morphology of Triconodontidae.

Confirmation of roll with the OFA contradicts two previous studies, which relied on jaw morphology and the corresponding musculature attachments for the modelling of jaw roll in Eutriconodonta, and that predicted little or no roll for Eutriconodonta^32,36^. Roll was linked to the presence of an angular process, which provides large muscular attachments for the M. masseter superficialis and M. pterygoideus medialis well below the central axis of the jaw^36^. Because an angular process is present in early Mammaliaformes such as *Morganucodon*, but absent in Eutriconodonta, the study predicted roll for the former and tentatively assumed that it was reduced in the latter. The second study similarly predicts little roll for Eutriconodonta based on jaw morphology and force vectors calculated for *Priacodon*^32^.

This discrepancy to the observed 10° of medially directed roll can be explained in two ways:

1. Roll during the power stroke was derived from musculature control but the previous hypotheses for the necessary jaw morphology/musculature arrangements are not correct. In that case the jaw of Eutriconodonta provided more leverage for the M. masseter superficialis than previously assumed. This could be due to the large masseteric fossa, which is positioned below the longitudinal axis of the jaw, albeit not as low as most angular processes. It provides a vertical surface so that the attachment of the M. masseter superficialis was dorsally placed and thus pulled vertically (supplementary information). This could make it easier to roll the jaw, compared to the attachment on a horizontal surface of an angular process.
2. Roll during the power stroke was a passive process in Eutriconodonta, thus jaw morphology is not a helpful indicator to predict the presence or absence of roll during occlusion. In this hypothesis the lower jaw rotated during the power stroke, after initial contact of the teeth, as a reaction to the orthally-directed pitching jaw movement and the inclined obstacle provided by the upper molars. The tips of the lower molar cusps entered the valleys of the uppers where they were redirected lingually. With continued upward-directed force the jaw would have tilted even without an appropriately-oriented muscular vector. This scenario is supported by the wear of the lower molar cusp tips (supplementary information). The part of the cusp tips closest to the center shows exposed dentine, while the buccal portion of the tip, although worn, has enamel still present. This suggests that the inner side of the lower cusp was subject to stronger attrition, as would occur during the first contact of the power stroke. This pattern is most noticeable on cusp a of m3 (Supplementary Fig. 4C,D) and matches the contact areas reproduced with the OFA (Supplementary Fig. 3).

We consider passive roll to be the more likely explanation, as it not only helps to explain why roll was predicted to be absent in triconodontids but also why no evidence for it was found in the occlusal analysis of *Morganucodon*^10^, despite being inferred based on jaw morphology^36^. Roll as a result, rather than an active process, in early mammals also supports the interpretation that the upper molars were inclined in the jaw to minimize roll in *Priacodon* as well as *Morganucodon*^10^. Therefore, the mobile symphysis and unilateral chewing of early mammaliaforms^37^ enabled the jaw to roll passively during the power stroke, thus resulting in a tighter fit and better matching occlusion. A similar thought was put forward on the possibility that minor medially and lingually directed roll during the power stroke of *Monodelphis* might have been caused by passive movement rather active musculature control^38^. Passive movement was disputed based on the observation of musculature controlled movements in *Monodelphis* in the active and inactive hemimandibles^39^. However, the morphology of the tribosphenic molar and the two-phased power stroke in therians are fundamentally different from observed and inferred conditions in most early mammals^28^. Molar morphology further supports the interpretation of passive roll (and yaw for that matter) for dentitions that rely on a single-phased power stroke^10,30^ (e.g. triconodonts, obtuse-angled “symmetrodonts”, dryolestids). Unless a directional change is required, as in tribosphenic or pseudotribosphenic dentitions, a vertically directed motion keeps the teeth in close contact, while the upper molars guide the power stroke. Any active medially directed roll or yaw would move the teeth away from each other, thus reducing contact and the capability to break down food.

### Dental function and diet

Several members of the Gobiconodontidae have been identified as carnivorous based on their size, dentition and stomach content^40,41^. Triconodontidae have been similarly interpreted as carnivorous^11,12^. Simpson^12^ even considered their dentition as “one of the most ideally carnivorous ever evolved”. However, this has been solely inferred from dental and jaw morphology because direct evidence, such as stomach contents or coprolites, is missing. Triconodontidae are also noticeably smaller than gobiconodontids, which include some of the largest known Mesozoic mammals^40,41^. Body mass in *Priacodon* can be estimated by reference to regressions based on dentary length^42^, estimated skull length^43^, and postcranial elements ^2,44^. These estimates range from 40.9–60.6 g in *P*. *fruitaensis*, to 111–175 g in *P*. *ferox* (supplementary information). Scaling upward, based on average molar lengths^13,45^, yields estimates of 253–375 g for the largest member of the family, *Jugulator amplissimus*. By way of comparison, these estimates place triconodontids among the smallest of living carnivorans, such as the least weasel (*Mustela nivalis*, 25–250 g) and the dwarf mongooses (*Helogale* spp., 230–680 g)^46^.

This raises the question as to what are the dental characters for the interpretation of a carnivorous diet for triconodontids? Carnivora have evolved the carnassials, where P4 and m1 form mesio-distally elongated cutting edges that pass along each other in close proximity. Homoplastic structures evolved in creodonts, Hyaenodontidae (M2/m3) and Oxyaenidae (M1/m2)^47,48^. Among carnivorous marsupials, *Thylacoleo carnifex* has greatly enlarged carnassial-like premolars^48,49^. Other marsupials such as *Thylacinus* lack specific carnassial teeth. However, all their molars have long, primarily mesio-distally oriented crests^47^, that enable the animal to slice portions of meat of larger prey.

Extant insectivorous taxa, on the other hand, often (though not exclusively) have molars with bucco-lingually oriented crests or lobes (e.g. zalambdodont or dilambdodont molars)^47,48^. Their dentitions provide a high amount of cutting edge length in total and are well suited for fragmenting small insects and food that fits into the mouth^50–52^, but due to their orientation lack the ability to cut off pieces from larger prey.

In Triconodontidae the cutting crests are oriented primarily mesio-distally^4^ (Fig. 2), supporting the interpretation that they were carnivorous^12^. However, the zigzag pattern of the upper molars forms additional bucco-lingually oriented crests. This unique combination of both properties provides the ability to fragment small prey (e.g. insects) into multiple small pieces with a single bite, which is not possible with a purely carnassial-like dentition. Thus, molar morphology of triconodontids is appropriate for carnivory, but also for fragmenting arthropods. Small- and medium-sized living predatory mammals are notoriously opportunistic in their feeding predilections, although the smallest species rely predominantly on insects and other arthropods, while progressively larger taxa incorporate more vertebrate prey into their diets^46,53^. Based on an estimated body mass range of ~ 40–375 g, a faunivorous diet combining insects and meat is appropriate for Triconodontidae, with the larger species relying more heavily on vertebrate prey. Also, later during ontogeny, the upper molars tend to lose their bucco-lingually oriented elements and are reduced to a straight mesio-distal crest^12^, as apparent in M1 of LACM 120451 (Fig. 1) (see below). This suggests a shift to a more carnivorous diet during ontogeny.

The occlusal function of the molar row in Triconodontidae has been compared to that of pinking shears, due to the zig-zag pattern of the upper molars^4^. Based on the OFA analysis, this comparison appears mostly correct. The lower molar crests pass along the upper molar crests in close proximity. With a straight upwards movement at the beginning of the power stroke prior to edge contact, the cusps of the lower molars penetrated the food with most of the initial bite force. After edge contact, the zig-zag patterns of the upper molars helped to sharpen the lower molars. Each lower cusp passed along an upper molar crest and subsequently entered a valley. Thus, the lower molars retained their pointed cusps and sharp crests. This explains why most upper molars of Triconodontidae show clear signs of wear and loss of relief^12^, while lower molars retain their relief well, even those of older individuals that exhibit extensive wear facets^54^.

Although pinking shears obviously do not rely on pointed cusps, they are a good analogue for the lower dentition of Triconodontidae, adding another functionality for food breakdown. This mechanism is also apparent in LACM 120451 although somewhat blurred by damage. Cusp a of m3 is pointed and sharp although the tip is worn and the dentine is exposed (supplementary information). Cusps C and D of M2 and cusp B of M3, on the other hand, retain a clear edge only on the buccal side (the main crest), while the rest of the occlusal surface is strongly worn with exposed dentine. This is even more pronounced in the more anterior molars since they have been longer in use. Despite having lost some of its tips by damage, it is apparent that cusp b of m2 is only little worn. On the corresponding distal part of M1, also damaged, most of the crown is worn down. The cusps are blunt and the valleys are shallow due to crown loss, and the former zig-zag edge of the lingual crest is almost completely straight due to wear.

### Comparison with early mammaliaform triconodont dentitions

Striations on the molars of the early mammaliaform *Morganucodon* have a high degree of variation, suggesting a considerable degree of freedom during occlusion^10^. This is in contrast to the parallel, uniform striation pattern seen in the molars of Triconodontidae (Supplementary Fig. 2), with the latter relying more on a precise uniform occlusion.

While the molar cusps of Triconodontidae are of equal size, the early mammaliaform triconodont pattern is characterized by a large main cusp A/a and smaller side cusps B/b and C/c. In *Morganucodon* this results in pronounced piercing at the beginning of the power stroke when only the main cusps are in contact^10^. In contrast, in *P*. *fruitaensis* the cusps of all molars came into contact in rapid succession and the upper and lower crests passed along each other in close proximity along the entire tooth row. While the latter applies also to the molar crests of *Morganucodon*, the available functional edge length was shorter than in Triconodontidae.

Jaw morphology, microtexture analysis, and molar morphology suggest that *Morganucodon* was able to prey on brittle insects by applying relatively large bite forces^10,55^.

Though the large masseteric fossa, angle of insertion of the superficial masseter, and robust jaw (supplementary information) suggest that *P*. *fruitaensis* and Triconodontidae in general were well adapted for powerful biting^12^, the dental morphology exhibits less emphasis on high bite forces.

The differences in molar morphology between Triconodontidae and early triconodont mammaliaforms suggest a functional change towards cutting rather than puncturing and shearing.

However, most taxa with *Morganucodon*-like dentitions were much smaller than the majority of Triconodontidae, with few exceptions such as *Paceyodon* or *Storchodon*^56,57^. Size must be considered when comparing the two molar types, because dental function is not only influenced by shape but also by scale^58,59^.

### Uniform dentition

The molar pattern of Triconodontidae changed little for approximately 65–85 ma from the Late Jurassic to the Late Cretaceous (Fig. 4). Cusp d becomes higher^15^, the tooth count increases, and the crown height increases, the latter being most apparent in the geologically youngest member *Alticonodon*^3^.

A possible explanation for the limited change is the constraints of the highly precise occlusion in a uniform tooth battery. As demonstrated by the OFA analysis, most molar cusps along the tooth row come into contact in rapid succession, with little freedom of movement provided by the close encompassment of the opposing valleys. This type of molar setting does not allow the development of new cusps or size changes in existing ones, since even small changes likely reduce the precise fit in such a uniform system. For comparison, a tribosphenic molar, or a plesiomorphic triconodont molar of stem mammaliaforms are more heterogeneous in cusp heights (Fig. 5), thus changes in size and shape of different cusps can more easily be integrated into the existing morphology with the potential to provide functional advantages. The homogenous shape of the molars of Triconodontidae thus constrained their possibility to evolve with major modifications. The little changes that occurred were within the constraints provided by the occlusion and maximized the total length of the cutting edges. Cusp d was already present in early Triconodontidae and was incorporated in the occlusion of the adjacent cusp c. Its gradual increase in size increased the length of the cutting edge of each molar without impairing the precise occlusal fit. This is apparent from *Astroconodon*, where cusp d is relatively large but remains closely associated with cusp c (Fig. 4)^13,15^. The increase in crown height exemplified by the lower molars of *Alticonodon* represents a similar mechanism. Although not adding to the maximum cutting edge length, it increased the amount of cutting edge length over the lifespan, without affecting occlusion. This increase of crown height might have been driven by the difference in the amount of wear in the upper and lower dentition, as discussed earlier. Apart from this, the addition of molars at the posterior end of the molar series was possible without interference with the constrained occlusal system. In *Triconodon* m4 is formed within the coronoid above the functional tooth row^54,60^. This unusual placement could be linked to an increase in tooth count, as the Early Cretaceous *Meiconodon* and the Late Cretaceous *Corviconodon* have five lower molars^13,60,61^.

## Conclusion

Triconodontidae exhibit a molar series that is unique among mammals and is not directly comparable to any extant counterpart. A highly homogenous cusp-valley system formed a continuous system of mesio-distally oriented crests that linked the entire molar series^12^. The occlusal mode of *P*. *fruitaensis* and probably Triconodontidae in general was embrasure occlusion. This is supported by premolar positioning, wear facets, and larger collision areas in the OFA analysis. This unifies embrasure occlusion as a plesiomorphic character for Eutriconodonta and limits the *Morganucodon* pattern to a few taxa within the Morganucodonta. The upper molars were inclined lingually within the maxilla, similar to Gobiconodontidae^2,8^ and *Morganucodon*^10,21^, which reduced the roll required for a matching occlusion. Roll of the active hemimandible during the power stroke was confirmed by the OFA analysis though it was likely a passive deviation of an upward jaw movement guided by the topographical structure of the upper molars. At the beginning of the power stroke, the mesio-distally oriented crests of the lower and upper molars passed along each other, and subsequently the lower cusps entered the valleys in between the upper cusps. Dental function of triconodonts thus differed from that of the early triconodont pattern of Morganucodonta, which relied more on puncturing capabilities of large cusp A/a rather than precise cutting. The dentition of Triconodontidae combines traits linked to carnivorous diets (e.g. mesio-distally oriented cutting edges) and insectivorous diets (bucco-lingually oriented crests and lobes). Therefore, their diet was likely faunivorous including insects and small vertebrates rather than fully carnivorous^11,12^. The loss of cutting edge length in the upper molars with increased wear is evidence for a change in diet during ontogeny. Based on the crest morphology, the pinking shear analogy^4^ holds up, but needs to be expanded to include the lower cusps, which are self-sharpening within the valleys of the upper molars.

The triconodontid molar series is highly uniform and adapted to a precise fit. While this ensured good cutting capabilities it likely put the dentition under greater evolutionary constraints than other molar types with more heterogeneous cusp morphologies.

## Material and methods

The holotype of *P. fruitaensis*, LACM 120451 (Natural History Museum of Los Angeles, formerly Los Angeles County Museum) comprises right and left partial dentaries and maxillae, parts of the skull, and associated postcranial material. The left ramus served as the basis for initial description^62^; subsequently, Rougier et al.^63^ provided an account of the petrosals, while Engelmann and Callison^64^ added important details to the dentary and lower dentition, also describing the maxillae and upper dentition, fragments of the skull, and some postcranial elements (humerus, radius, and partial femur). For this study, the right ramus and maxilla were re-examined (Fig. 1; Supplementary Fig. 6). The anterior part of the ramus is missing, p4, m1-4 are preserved but several cusps are damaged and p4 was shifted upwards and is not in its natural position. The condyle and the posterior part of the ramus are complete, with the exception of the uppermost region of the ascending ramus. The maxilla contains four premolars and three slightly damaged molars. Kielan-Jaworowska et al.^4^ present a restoration of the skull of *Priacodon*, based on morphology of LACM 120451.

Existing hypotheses on the occlusion were virtually tested with the Occlusal Fingerprint Analyser (OFA) software (ZiLoX IT GbR; Occlusal Fingerprint Analyser; Version: 1771 × 86_64 https://www.ifgeo.uni-bonn.de/en/ifg_homepage/departments/paleontology/labs/vertebraten/ehemalige-forschergruppen/for-771/ofa). The term ‘occlusal fingerprint’, describes the orientation and position of wear facets on the occlusal surface^65^. The OFA software was developed within the Research Unit 771 of the German Research Foundation (DFG) and is used to virtually analyze the chewing path and to test hypotheses on occlusal relationships^25,30,64–70^. For the OFA analysis, the specimen was scanned with micro-computed tomography (µCT), using an NSI scanner at the University of Texas High-Resolution X-ray CT facility, with the following parameters: Fein Focus High Power source, 110 kV, 0.15 mA, no filter, Perkin Elmer detector, 0.25 pF gain, 1 fps, 1 × 1 binning, no flip, source to object 137.092 mm, source to detector 1316.876 mm, continuous CT scan, 2 frames averaged, 0 skip frames, 2400 projections, 5 gain calibrations, 0.762 mm calibration phantom, data range [− 10.0, 500.0] (grayscale adjusted from NSI defaults), beam-hardening correction = 0.275. Voxel size = 10.4 μm. Total number of slices = 1934. Subsequently, the scan was segmented and a virtual 3D model was created using Avizo 8.1 (Visualization Sciences Group, France). Some data processing, e.g. virtual alignment and the reduction of triangles, was performed with Polyworks (2014, InnovMetric Software Inc., Canada); file format is .stl (little endian). For the OFA analysis, reduced models of m2–m4 and M2 and M3 were used. The anterior teeth were left out due to damage, which made them unsuitable for the analysis. Previous studies tested competing occlusal hypotheses by OFA analysis^25^. Accordingly, the collision distance during the power stroke was used to detect the best-fit hypothetical path. Further, the OFA was used to simulate roll (medial tilting of the dentary along its anterior–posterior axis) during the power stroke^25^.

Values presented here for the roll rate refer to the degree of medial tilting during the power stroke. 0° is defined as the orientation in which the transverse axis of the condyle is in a horizontal position and the upper and lower premolars are oriented straight vertically.

The dental striations of *P*. *fruitaensis* were examined with scanning electron microscopy (SEM) (Cambridge CamScan MV2300) of a cast. The cast was made in polyurethane resin (BJB Enterprises, product TC-892).

For comparison of the molar complexity (Fig. 5), a molar of an undescribed triconodontid from the Cloverly Formation, an m2 of *Morganucodon watsoni* (UMZC Eo.CR.1) and a m1 of *Cantius ralstoni* (USGS 13634) were compared.

For comparison of the striation (Supplementary Fig. 2), casts of a lower molar of A*stroconodon denisoni* (SMP SMU 61759) and *Arundelconodon hottoni* (USNM 491129) were examined.

For comparison of the posterior ramus (Supplementary Fig. 7), two specimens of *Triconodon mordax* (NHMUK PV OR 47764; NHMUK PV OR 47763) and a specimen of *Trioracodon ferox* (NHMUK PV OR 47775), from the Natural History Museum, London were examined.

## Supplementary Information

Supplementary Information 1.

Supplementary Information 2.

Supplementary Information 3.

Supplementary Information 4.

Supplementary Information 5.

Supplementary Information 6.

Supplementary Information 7.

Supplementary Information 8.

Supplementary Information 9.

Supplementary Information 10.

Supplementary Information 11.

Supplementary Information 12.

Supplementary Information 13.

Supplementary Information 14.

## Data Availability

3D Data presented in this study is available via Morphosource: https://www.morphosource.org/index.php/Detail/MediaDetail/Show/media_file_id/159178.
