# Supplementary material for: Molar occlusion and jaw roll in early crown mammals
Source: Sci Rep. 2020 Dec 24;10:22378. doi: 10.1038/s41598-020-79159-4 (PMC7759581; doi:10.1038/s41598-020-79159-4)
Supplement: Supplementary file 1 — Supplementary Information 1. [file 41598_2020_79159_MOESM1_ESM.docx]

**Supplementary Information**

**Molar Occlusion and Jaw Roll in Early Crown Mammals**

by *KAI R. K. JÄGER^1^, RICHARD L. CIFELLI^2^, and THOMAS MARTIN^1^

^1^Section Palaeontology, Institute of Geosciences, Rheinische Friedrich-Wilhelms-Universität Bonn, Nussallee 8, 53115 Bonn, Germany; e-mails: [jaegerk@uni-bonn.de](mailto:jaegerk@uni-bonn.de); tmartin@uni-bonn.de

^2^Oklahoma Museum of Natural History, 2401 Chautauqua Ave, Norman, OK 73072 USA; e-mail: RLC@ou.edu

**Taxonomy of Triconodontidae**

In this study we follow the definition for Triconodontidae of Gaetano and Rougier (2011) as “the less inclusive group containing *Priacodon* and *Triconodon*, their common ancestor and all its descendents” [sic]. Triconodontidae have been reported from North America, Europe, Asia, and possibly South America (Cifelli *et al*. 1998; Rougier *et al.* 2007a, 2007b; Kusuhashi *et al*. 2009).

Most of the uncontested triconodontid taxa are of Late Jurassic or younger age. However, some fossils suggest an earlier diversification of the family. Montellano *et al*. (2008) described a potential early triconodontid from the late Early Jurassic of Mexico, *Victoriaconodon*, which shares some of the premolar characters found in Triconodontidae although the molars retain presumptively primitive cusp proportions (cusp a notably taller than b and c). Of similar age is the South American *Argentoconodon*, which shows similarities to *Volaticotherium* and *Ichthyoconodon*. These three taxa may represent a clade of derived gliding Triconodontidae (Gaetano and Rougier 2011). If these taxa indeed belong to Triconodontidae, one of the most derived representatives of the family (*Argentoconodon*) would be the oldest known so far. However, according to a recent phylogeny by Martin *et al.* (2015), *Argentoconodon* and *Volaticotherium* fall outside of Triconodontidae. Another potential early representative is *Eotriconodon* from the Bathonian of the United Kingdom (Butler and Sigogneau-Russell 2016). Here we follow the phylogeny provided by Martin *et al.* (2015).

The three most plesiomorphic taxa of Triconodontidae are *Priacodon*, *Triconodon*, and *Trioracodon* (Gaetano and Rougier 2011, Martin et al. 2015) from the Late Jurassic and Early Cretaceous of the United Kingdom and the United States, respectively (Simpson 1925a, 1925b, 1928, 1929). With highly similar molar morphologies, tooth count is the most reliable character to distinguish between these taxa.

The differences in premolar and molar count are: *Triconodon* P?/4 M?/4, *Priacodon* P3/3 M4/4, and *Trioracodon* P4/4 M3/3. However, tooth count between the different genera is also problematic, because upper and lower tooth counts can be different. In *P*. *fruitaensis*, for example, the postcanine formula is P4/3 and M3/4 (Engelmann and Callison 1998).

**Morphology of the Ramus and Muscle Insertions**

The dentaries of *Priacodon* *fruitaensis* have previously been described in detail (Rasmussen and Callison 1981, Engelmann and Callison 1998). Here we confine our observations to additional information from the µCT data and to characters that directly influenced the occlusion. The most noticeable character of the sturdy ramus is the wide masseteric fossa on the distolateral side (Engelmann and Callison 1998), bordered ventrally by a prominent crest or flange. In LACM 120451 its size is slightly exaggerated because the ventrodistal part of the ramus is broken and shifted medially. Simpson (1933) provided a schematic cross-section of the ramus of *Priacodon* (species not indicated) with a pronounced masseteric fossa, commenting that it is less strongly developed than that of *Triconodon*. According to the µCT data, the masseteric fossa of *P*. *fruitaensis* is larger than that of both *Triconodon* and *Trioracodon*, with a more pronounced masseteric crest, even though *P*. *fruitaensis* is of similar size as *Triconodon* and smaller than *Trioracodon* (Supplementary Fig. 7).

The importance of the masseter muscle for *P*. *fruitaensis* is also highlighted by a large masseteric foramen in the anterior ventral wall of the masseteric fossa. This foramen is present on both rami and was originally referred to as blind ending “pocket” or “pit” (Rasmussen and Callison 1981, Engelmann and Callison 1998). The foramen is, however, connected to the mandibular canal (Supplementary Fig. 6) and likely transmitted a blood vessel supplying the large masseter muscle. Engelmann and Callison (1998) further noted that this foramen (“pocket” or “pit”), although not mentioned by previous authors, is present in all “triconodont specimens” from Como Bluff. It has also been described for several other Triconodontidae, e.g. *Corviconodon utahensis, Astroconodon denisoni*, *Priacodon ferox*, *Triconodon mordax* and *Trioracodon bisulcus* (Cifelli et al. 1998), as well as *Vincelestes*, some early therians, and the Recent seal *Phoca* (Cifelli and de Muizon 1997, Davis 2012). A notable similarity to the foramen described here and the large masseteric foramen reported for *Peramus* is that in both cases the connection to the mandibular canal is at the position of m3 (Supplementary Fig. 6) (Davis 2012 Fig. 3B).

The condyle of LACM 120451 is slightly damaged but noticeably wider than long and curved upwards (Fig. 1). The damaged articular surface is oriented posterodorsally. It is oval (transversely elongated) and the anterior margin is convex anteriorly (Engelmann and Callison 1998), being similar to that of other Triconodontidae such as *Trioracodon* (Simpson 1928).

**Posterior Mandibular Ramus and Adductor Musculature Reconstruction**

The adductor musculature was reconstructed for *Priacodon fruitaensis*, *Trioracodon ferox* and two specimens of *Triconodon mordax*. Discernible on the dorsolateral side of the coronoid process of all taxa is a large area that probably marks the insertion of the *M. temporalis*. In *Triconodon* and *Trioracodon* it is separated from the region where the tendons for the *M. masseter profundus* inserted by a slight protuberance approximately at the height of the neck (Supplementary Fig. 7). In *P*. *fruitaensis* this separation is not visible, thus the position of the *M. temporalis* is tentatively assigned based on its position in the other specimens.

There is no clear morphological border that separates the attachment surfaces for the *M. masseter profundus* and *M. masseter superficialis*. However, since this division was already present in non-mammalian synapsids and more plesiomorphic mammaliaforms such as *Docodon* and *Morganucodon,* it is highly likely that this division was also present in *Triconodon* (Lautenschlager *et al.* 2017; Schultz *et al.* 2019). We consider the most likely attachment for the *M. masseter superficialis* to be on the ventral side of the wide masseteric fossa, extending toward the masseteric crest (sensu Simpson 1928). It is noteworthy that Simpson (1928a) described a pterygoid crest “Pt.C.” that extends ventral to the masseteric crest on the lateral side of the dentary (Simpson 1928a, fig. 28). This is puzzling, given that both the medial and lateral pterygoid muscles insert on the medial side of the dentary in mammals, and it is in contrast to his own use of the designation “Pt.C” for a ridge on the medial side of the dentary in *Docodon* (Simpson 1928a, fig. 3). Additionally, in the holotype of *Trioracodon* *ferox* (PV OR 47775) contrary to the drawing by Simpson (1928a, fig. 28) there is no evidence for a crest or surface ventral to the masseteric crest that is visible in lateral view (Supplementary Fig. 7B). Thus, the masseteric crest demarcates the ventral boundary of the posterior mandibular ramus (Supplementary Fig. 7).

The medial side of the coronoid process of all taxa examined has a slight depression that functioned as an attachment for the *M. temporalis*. This is in accordance with extant mammals, as well as reconstructions of musculature arrangements of non-mammalian Mammaliaformes and Mesozoic Mammalia (Lautenschlager *et al.* 2017, Schultz *et al.* 2019). The insertion of *M. temporalis* most likely also covered the crypt of the developing m4. The areas of attachment for the *M. pterygoideus lateralis* and *M. pterygoideus medialis* are tentatively assigned. The ambiguity for the former arises from the morphological differences between the specimens. Anterior to the condyle, *P*. *fruitaensis* and the holotype of *Triconodon mordax* (PV OR 47764) have a slightly inclined, almost flat surface for the attachment of the *M. pterygoideus lateralis*. This area in PV OR 47763 is considerably steeper and less separated (Supplementary Fig. 7D). In *Trioracodon* a flat surface as well as a lateral one is present, but the surface of the latter appears to be accentuated, thus the position of the lateral pterygoid muscle is assigned tentatively.

The attachment area for the *M. pterygoideus medialis* is ambiguous. Small surfaces just ventral to the M. temporalis are clearly noticeable in all specimens and are tentatively considered the most likely insertion surfaces. However, PV OR 47763, PV OR 47764 and PV OR 47775 do also show a potential insertion surface on the ventral side of the ramus. In PV OR 47763 and PV OR 47775 this surface is circumvented by a bulged margin. Given the different orientation and size of both potential regions, they hold noticeable implication for the size and importance of the *M. pterygoideus medialis*.

**Wear Facets**

**Lower dentition**

p4

With the exception of apical wear, there are no distinct wear facets on p4. The anterior flank of cusp a shows a slightly rugged surface which is difficult to interpret. It might be a wear facet that was damaged post-mortem; however, given the lack of damage on the rest of the tooth, we provisionally consider this region as lacking a wear facet.

m1

The mesiobuccal sides of cusps b and c have clearly defined, large wear facets. The only difference is that the facet on cusp c is oriented slightly more buccally than the facet on cusp b. Cusp a differs from b and c in having its enamel still preserved, except for the worn tip. A clearly separated facet cannot be distinguished on cusp a, but many similarly oriented striations are visible on the enamel surface, and the enamel appears to be thinner on the buccal side (the occlusal side of lower molars) than on the lingual side. This suggests that attrition (tooth-tooth contact) was present and equally distributed on cusp a.

m2

Most of the surface of m2 is damaged. The best-preserved cusp is b. The buccal and distal sides of cusp b show signs of wear with polished enamel. The most mesial part of cusp b has dentine exposed. Dentine probably was exposed on the tip as well, but this remains unclear due to damage. The valley between cusps a and b appears to have lost more of its enamel than the adjacent cusps. Due to damage little can be said regarding wear facets on cusps a and c, except that polished enamel is present.

m3

The m3 is well preserved with clear wear facets (Supplementary Fig. 4). The mesiobuccal region of cusp b has a wear facet with exposed dentine similar to cusp b of m1 and m2. Dentine is also exposed on the tip, which results in a prominent step to the enamel crest on the top of cusp b. The enamel on the buccal side is polished. On the distobuccal flank of cusp b the enamel is polished and partially worn away. This facet expands into a dentine depression between cusps a and b, near the base of the crown. The exposure of dentine so low on the crown, while enamel is still present higher on the cusp flank, could have been caused by increased wear near the base of the tooth or by thinner enamel in that region. The tip of cusp a is well preserved and exhibits a combination of polished enamel and exposed dentine that was likely also present on the damaged tip of cusp b, given the similarity to the preserved wear on cusp b. On the tip of cusp a, a sharp enamel edge borders deeply excavated dentine. Based on the excavated dentine it is evident that this edge was the leading edge where contact to the antagonist was first initiated (Greaves 1973; Rensberger 1973; Costa and Greaves 1981). The exposed dentine extends along the mesiobuccal flank into the valley between cusps a and b. From the tip in a buccal direction, the dentine is not exposed and enamel is still preserved, resulting in a second, smoother trailing edge. This enamel facet follows the same mesiobuccal inclination as the exposed dentine (Supplementary Fig. 4). These observations suggest that, during the initial contact of the cusp tips at the beginning of the power stroke, most of the attrition forces were oriented mesially into the valley. Thus, the enamel on the buccal side of cusp a is still preserved while on the mesial side it is already worn away. This is evidence for a distal component in the orthal movement of the power stroke, which is further supported by the orientation of the striations and the OFA analysis (see below).

An enamel facet is present on the distal side of cusp a, which merges into a dentine facet in the valley between cusps a and c. Although the tip of cusp c is damaged, dentine is exposed on its mesial flank and is bordered buccally by polished enamel, suggesting a similar condition to that observed on the mesiobuccal flank of cusp a. On the distobuccal flank of cusp c a wear facet is present with exposed dentine that extends into the valley between cusp c and cusp d. Compared to the other valleys, the dentine does not extend so far down before the facet ends in a shallow trailing edge to the enamel. Cusp d is enveloped by the mesial interlocking embayment of m4, and therefore fully incorporated in its mesial facet, with its dentine exposed as well.

m4

Like cusp a of m3, the mesial side of cusp b bears a large wear facet with exposed dentine that extends mesially. On its buccal side enamel is still present but is polished. The distobuccal side of cusp b bears a wear facet that differs from the homologous facets on the anterior molars because it does not extend deeply into the valley between cusps a and b but ends at the base of cusp b. Similarly, the mesial facet on cusp a apparently does not extend into the valley, but this is ambiguous due to damage. The most posterior facet is a small, distally-oriented one on the upper distobuccal side of cusp a, with polished enamel. Cusp c is unworn.

**Upper dentition**

P4

The P4 shows few clearly developed wear facets. The most distinctive is a small facet at the cingulum on the lingual base of cusp C. The dentine is exposed, showing some faint striations, and the enamel edge is polished, which suggest that this facet was likely caused by attrition. Compared to the wear stage indicated by the molars, the facet is small. A faint wear facet extends from the posterior flank of cusp A to the tip of cusp C. It yields no clear picture under the SEM due to corrosion or coverage (such as glue or a consolidant resin) of some sort. A wear facet is tentatively assigned to this region, since cusp A shows apical wear that widens distally and matches the ambiguous area. The tip of cusp A is worn. Dentine is exposed on the anterior and buccal sides and is surrounded by an enamel ring. From there, polished enamel extends slightly down on the distolingual side. While ambiguous, we think that the area in question shows an incipient wear facet. The anterior side of P4 exhibits striations but lacks distinct wear facets.

Given the wear on the molars the lack of wear on both the upper and lower ultimate premolars is surprising. One potential explanation is that these teeth had been recently replaced. However, in *Triconodon* p4 is replaced before the eruption of the last lower molar (Jäger *et al.* 2020). If a similar timing is assumed for *P*. *fruitaensis*, wear facets on the premolars would be expected. The lack of wear on the ultimate premolars thus might indicate different timing of the eruption sequence in *P*. *fruitaensis*.

M1

The wear on M1 mostly remains ambiguous since the tooth is damaged. It is unclear if dentine exposure in some regions was caused by wear or post-mortem damage. The valley between cusps A and C shows exposed dentine as well as polished enamel. This is similar to M2 and M3 and is characteristic for wear caused by the cusp tips of the antagonistic lower molars. The posterior side of cusp C exhibits polished dentine with faint striations, which is also the case in M2 and M3. The enamel appears to be less polished than on the posterior molars, probably owing to post-mortem damage. Between cusps B and A dentine is exposed and apparently most of cusp B was worn away, although this is not quite clear due to damage. Therefore, a wear facet is tentatively assigned to the distolingual flank of cusp B.

M2

M2 is well preserved and the facets are clearly recognizable. Most of the lingual side is affected by attrition, with four regions showing clear distinct features: The anterior flank of cusp B, the valley between cusps B and A, the valley between cusps A and C, and the posterior flank of cusp C. These regions exhibit polished enamel and dentine with parallel striations. Unlike the valleys, the cusp tips are worn down to blunt ridges between the valleys and striations there tend to be less pronounced. The height differences between the leading edges of the crests and the trailing edges close to the crown base are less pronounced than in the lower molars.

M3

The anterior flank of cusp B, the valley between cusps B and A, the valley between cusps A and C, as well as the tip of cusps B and A show similar wear patterns as described for M2. The only difference in wear to M2 is on the posterior flank of cusp C. Unlike on the anterior molars, this flank lacks a facet since it did not come into contact with the antagonistic m4. The posterior part of the tip of cusp C, however, shows signs of wear. This region is somewhat affected by damage, which makes it difficult to identify the properties of the facet. However, a circular exposure of dentine and enamel is recognizable. The latter becomes smoother towards the base (the direction an antagonist would have travelled) and shows remnants of striations. As the most posterior facet on the upper molars, it is likely that it was in contact with the most posterior facet on cusp a of m4. Given its size relative to the small cusp a of m4, the worn tip of cusp C must have almost encircled the smaller lower cusp. Therefore, the alternating pattern of cusp-valley occlusion was lost on the most posterior molars and replaced by direct cusp-cusp attrition.

**Maxillary pits**

In addition to the wear facets, maxillary pits that occur lingually to the upper teeth can provide important information for the reconstruction of the occlusion. Unfortunately, only the anterior part of the maxillary palatal plate is preserved. A single pit is present lingual to the M1 (Fig. 1). Its location and depth are puzzling for several reasons. The a cusps of the lower molars are only slightly larger than their neighbours, yet the depth of the pit is roughly comparable to those present in *Morganucodon*, which has much larger main cusps (Jäger et al. 2019). Also, no more anteriorly situated pit is present, despite the large size of p4. The deepest part of the pit sits lingually to cusp C of M1. When these observations are taken into account and the 3D models are set in a late occlusal position, the position of the pit matches better with Simpson´s occlusal model than the one proposed by Mills.

**Striation analysis**

SEM images of the wear facets of *P*. *fruitaensis* show a primarily orthal orientation of striations with a slight posterior component (Supplementary Fig. 2). Striations are present on the enamel and, less clearly, on the dentine. On wear facets formed by attrition, most striations are parallel.

Striations on areas without clearly defined wear facets tend to be less regular, with individual deep scratches showing a wider variety of orientations. Nevertheless, the majority of the striations is oriented orthally with a minor posterior deviation at around -5° (with 0° representing straight orthal orientation). Similar parallel striations are present on the molars of the Cretaceous triconodontids *Astroconodon denisoni* and *Arundelconodon hottoni* which are slightly more vertically oriented, with orientations of approximately -2°. Striations described for *Corviconodon utahensis* are inclined further distally than those of *P*. *fruitaensis* and *Astroconodon denisoni* (Cifelli and Madsen 1998). This difference is independent of the molar position, and it is more likely that the inclination of the power stroke of *Corviconodon* *utahensis*, with approximately -15°, was more posteriorly directed than that of the other taxa described here. However, given the uniform striation pattern and little freedom provided by the molar morphology, it is safe to assume a very consistent power stroke with little variation for all Triconodontidae. This contrasts with striations on molars of *Morganucodon watsoni*. In this early mammaliaform striations can vary from -20° to +20°, sometimes within a single specimen (Jäger *et al.* 2019).

Striations on LACM 120451 are better visible on the lower than on the upper molars. This is likely due to stronger damage of the upper molars and the concentration of the upper molar striations in the valleys between the cusps, which makes a detailed SEM examination more difficult.

**Body Mass in *Priacodon***

**Estimates based on dentary length**

Foster (2009) estimated body mass for mammals of the Morrison Formation based on dentary lengths. He reported estimates of **110.5g** and **115.4** g for *Priacodon*, using regressions for (1) modern marsupials alone; and (2) marsupials plus insectivorans, respectively; and a dentary length of 33 mm from Simpson (1929). Simpson’s restoration (Simpson 1929, Fig. 11) is based on YPM 606 (holotype of the type species *P*. *ferox*), which is complete except for the condyle and posterior part of the coronoid process (Marsh 1887 plate X, fig. 9).

The left dentary of *Priacodon fruitaensis* (LACM 120451), which is also missing part of the coronoid process but is complete from condyle to the anteromesial end of the symphysis, measures 25.6 mm (Rasmussen and Callison 1981). Application of Foster’s regressions yields the following results:

1. Ln (Body Mass) = 2.9677 (ln Dentary Length) – 5.6712 = **52.0 g**
2. Ln (Body Mass) = 2.5344 (ln Dentary Length) – 4.1134 = **60.6 g**

**Estimates based on skull length**

Skull length has been used to calculate body mass in stem mammals (non-mammalian mammaliaforms, including *Sinoconodon*, *Morganucodon*, and *Hadrocodium*), which, like triconodontids, have a triconodont molar pattern (Luo et al. 2001). The regression of Luo et al. (2001, fig. 5B), based on skull length in a sample of 37 extant insectivores, is: log_10_ (Body Mass) = 3.68 (log_10_ Skull Length) – 3.83. The most recent restoration of the skull in *Priacodon*, which incorporates morphology of both *P*. *ferox* and *P*. *fruitaensis* but is based on measurements of the type species, is that of Kielan-Jaworowska et al. (2004, fig. 7.3A). The skull and dentary lengths in this restoration are 44.7 and 36.4 mm, respectively (the dentary being slightly longer than in the estimate used by Foster (2009). Applying the regression of Luo et al. (2001), a body mass estimate for *Priacodon ferox* based on skull length is **175.0 g**. Scaling skull length to dentary length(44.7 / 36.4 = 1.2280) and using the actual length of the dentary in *P*. *fruitaensis* (25.6 mm) to estimate length of the skull, we get an estimated skull length of 31.4 mm for the species. Applying the regression of Luo et al. (2001) yields the following body mass estimate for *Priacodon fruitaensis*:

Log_10_ (Body Mass) = 3.68 (log_10_ Skull Length) – 3.83 = **47.9 g**

This result is close to the estimate of 52.0 g based on Foster’s primary regression using dentary length.

**Estimates based on humerus**

Campione and Evans (2012) demonstrated that circumference of proximal limb elements (humerus, femur) is strongly correlated with body mass in extant mammals and reptiles, despite differences in posture, locomotion, and phylogenetic relationships. The holotype of *Priacodon* *fruitaensis*, LACM 120451, includes a nearly complete left humerus. We obtained three estimates of body mass in *P*. *fruitaensis*, using the least circumference of this element (4.5 mm) and regressions calculated by Campione and Evans (2012) from three samples of extant vertebrates:

1. Reptiles + Mammals (234 taxa)

Log_10_ (Body Mass) = 2.6861 (Log_10_ Humerus Circumference) – 0.1438 = **45.9 g**

1. Mammals (200 taxa)

Log_10_ (Body Mass) = 2.6938 (Log_10_ Humerus Circumference) – 0.1655 = **44.2 g**

1. Mammals < 20 kg (138 taxa)

Log_10_ (Body Mass) = 2.7768 (Log_10_ Humerus Circumference) – 0.2550 = **40.9 g**

These estimates are less than values calculated from measurements of the skull and dentary, and raise an interesting point: mammals with a triconodont molar pattern have relatively larger skulls and jaws than do modern mammals, reflecting a relatively larger feeding apparatus. By way of illustration, the humerus of *Priacodon* *fruitaensis* is only 14.5 mm long, or less than half the estimated skull length, and 1.4 mm in least diameter, or slightly more than half the length of a lower molar. Skull and skeletal proportions are similar in the Cretaceous gobiconodontid *Spinolestes* *xenarthrosus*, for which body mass estimates vary in a similar fashion (Martin et al. 2015). The relatively large feeding apparatus in triconodonts suggests the possibility that they may have fed on relatively larger prey than do extant mammals of similar body size.

Body mass in *Jugulator*

The largest triconodontid is *Jugulator* *amplissimus*, from the Cenomanian (early Late Cretaceous) of central Utah (Cifelli and Madsen, 1998). *Jugulator* is known only by isolated teeth, and hence its body mass can only be estimated indirectly, using the common procedure of multiplying the mass of an established standard by the cube of a scaling factor, based on comparable linear measurements of the unknown and established standard (e.g., Colbert, 1962). Here we use *Priacodon* *fruitaensis* as the standard for comparison, employing average length of non-first lower molar (2.55 mm for *P*. *fruitaensis*, Rasmussen and Callison 1981; 4.68 mm for *J*. *amplissimus*, Cifelli and Madsen 1998, table 1) for scaling, and minimum (1) and maximum (2) mass estimates from above, in the calculations:

1. *Jugulator amplissimus*, Body Mass Minimum = (4.68 / 2.55)^3^ * 40.9 g = **252.8 g**
2. *Jugulator amplissimus*, Body Mass Maximum = (4.68 / 2.55)3 * 60.6 g = **374.6 g**

**References**

Barghusen, H. 1968. Mechanics and the evolution of the synapsid jaw. *Evolution*, **26**, 622-637.

Butler, P.M. and Sigogneau-Russell, D. 2016. Diversity of triconodonts in the Middle Jurassic of Great Britain. Palaeontologia Polonica 67: 35–65.

Campione, N.E. and Evans, D.C. 2012. A universal scaling relationship between body mass and proximal limb bone dimensions in quadrupedal terrestrial tetrapods. *BMC Biology* 10 (60): 1–21.

Cifelli, R.L. and Madsen, S.K. 1998. Triconodont mammals from the medial Cretaceous of Utah. Journal of Vertebrate Paleontology 18 (2): 403–411.

Cifelli, R.L. and Muizon, C. de. 1997. Dentition and jaw of *Kokopellia* *juddi*, a primitive marsupial or near-marsupial from the medial Cretaceous of Utah. Journal of Mammalian Evolution 4 (4): 241–258.

Cifelli, R.L., Wible, J.R., and Jenkins, F.A., Jr. 1998. Triconodont mammals from the Cloverly Formation (Lower Cretaceous), Montana and Wyoming. Journal of Vertebrate Paleontology 18 (2): 237–241.

Costa, R.L., Jr. and Greaves, W.S. 1981. Experimentally produced tooth wear facets and the direction of jaw movements. Journal of Paleontology 55: 635–638.

Davis, B.M. 2012. Micro-computed tomography reveals a diversity of peramuran mammals from the Purbeck Group (Berriasian) of England. *Palaeontology* 55: 789–817.

Engelmann, G.F. and Callison, G. 1998. Mammalian faunas of the Morrison Formation. *Modern Geology* 23: 343–379.

Foster, J.R. 2009. Preliminary body mass estimates for mammalian genera of the Morrison Formation (Upper Jurassic, North America). *Paleo Bios* 28 (3): 114–122.

Gaetano, L. and Rougier, G. 2011. New materials of *Argentoconodon fariasorum* (Mammaliaformes, Triconodontidae) from the Jurassic of Argentina and its bearing on triconodont phylogeny. *Journal of Vertebrate Paleontology* 31: 829–843.

Greaves, W. 1973. The inference of jaw motion from tooth wear facets. *Journal of Paleontology* 47: 1000–1001.

Jäger, K. R. K., Gill, P. G., Corfe, I., and Martin, T. 2019. Occlusion and dental function of *Morganucodon* and *Megazostrodon*. *Journal of Vertebrate Paleontology* e1635135: 1–21. DOI:10.1080/02724634.2019.1635135.

Jäger, K. R. K., Cifelli, R. L., and Martin, T. 2020. Tooth eruption in the Early Cretaceous British mammal *Triconodon* and description of a new species. *Papers in* *Palaeontology* 2020:1-16 doi: 10.1002/spp2.1329

Kielan-Jaworowska, Z., Cifelli, R.L., and Luo, Z.-X. 2004. *Mammals from the Age of Dinosaurs: Origins, Evolution and Structure*. 630 pp. Columbia University Press, New York.

Kusuhashi, N., Hu, Y., Wang, Y., Hirasawa, S., and Matsuoka, H. 2009. New triconodontids (Mammalia) from the Lower Cretaceous Shahai and Fuxin formations, northeastern China. Geobios 42: 765–781.

Lautenschlager, S., Gill, P. G., Luo, Z.-X., Fagan, M. J., and Rayfield, E. J. 2017. Morphological evolution of the mammalian jaw adductor complex. *Biological Reviews* 92:1910–1940.

Luo, Z.-X., Crompton, A.W., and Sun, A.-L. 2001. A new mammaliaform from the Early Jurassic and evolution of mammalian characteristics. *Science* 292: 1535–1540.

Marsh, O.C. 1887. American Jurassic mammals. *American Journal of Science* 33: 326–348.

Martin, T., Marugán-Lobón, J., Vullo, R., Martín-Abad, H., Luo, Z.-X., and Buscalioni, A.D. 2015. A Cretaceous eutriconodont and integument in early mammals. *Nature* 526: 380–385, doi:10.1038/nature14905.

Montellano, M., Hopson, J.A., and Clark, J.M. 2008. Late Early Jurassic mammaliforms from Huizachal Canyon, Tamaulipas, Mexico. Journal of Vertebrate Paleontology 28 (4): 1130–1143.

Rasmussen, T.E. and Callison, G. 1981. A new species of triconodont mammal from the Upper Jurassic of Colorado. *Journal of Paleontology* 55: 628–634.

Rensberger, J.M. 1973. An occlusion model for mastication and dental wear in herbivorous mammals. *Journal of Paleontology* 47: 515–528.

Rougier, G.W., Garrido, A., Gaetano, L., Puerta, P., Corbitt, C., and Novacek, M.J. 2007a. First Jurassic triconodont from South America. *American Museum Novitates* 3850: 1–17.

Rougier, G.W., Isaji, S., and Manabe, M. 2007b. An Early Cretaceous mammal from the Kuwajima Formation (Tetori Group), Japan, and a reassessment of triconodont phylogeny. *Annals of the Carnegie Museum* 76: 73–115.

Simpson, G.G. 1925a. Mesozoic Mammalia. I. American triconodonts, part 1. American Journal of Science 10: 145–165.

Simpson, G.G. 1925b. Mesozoic Mammalia. I. American triconodonts: part 2. American Journal of Science 10: 334–358.

Simpson, G.G. 1928. A Catalogue of the Mesozoic Mammalia in the Geological Department of the British Museum. 215 pp. Trustees of the British Museum, London.

Simpson, G.G. 1929. American Mesozoic Mammalia. Memoirs of the Peabody Museum 3 (1): 1–235.

Simpson, G.G. 1933. Paleobiology of Jurassic mammals. Palaeobiologica 5: 127–158.

Schultz, J.A., Bhullar, B.-A., and Luo, Z.-X. 2017. Re-examination of the Jurassic mammaliaform *Docodon victor* by computed tomography and occlusal functional analysis. *Journal of Mammalian Evolution* 26 (1): 9–38.

Turnbull, W. D. 1970. Mammalian masticatory apparatus. *Fieldiana: Geology*, **18**, 149-356.

Supplementary Figure 1

Alternative interpretations of the postcanine occlusion of *P*. *fruitaensis* (right maxilla and mandible in lateral view). A, after Simpson (1925) and B, after Mills (1971). Squares highlight differences in the anterior and posterior part of the tooth row. The occlusion after Mills (1971) results in the ultimate premolars not interlocking and the m4 left without an antagonist. The SEM images show wear facets on the m4 and m1 that after Simpson (1925) require occlusion to form. Note that p4 was repositioned in the virtual model to its natural position since it was post mortally damaged and shifted upwards.

Supplementary Figure 2

SEM images of molars of different Triconodontidae. A, isolated left lower molar of *Astroconodon* *denisoni* in lingual and occlusal view (SMP SMU 61759); B, right m2 of *Arundelconodon hottoni* in lingual view (USNM 491129); C, right m3 of *P*. *fruitaensis* in lingual and occlusal view; D, right of *P*. *fruitaensis* in lingual view; E, M3 of *P*. *fruitaensis* in occlusal view; F, M4 of *P*. *fruitaensis* in occlusal view. Striations in all taxa indicate a steep, single phased power stroke. The posterior facet of m4 in *P*. *fruitaensis* (D) corresponds to the posterior facet of M4 (F). Mesial is to the left in A and to the right in B-F. Scale bar equals 1 mm.

Supplementary Figure 4

Corresponding wear facets of the right M3 and right m3 of the holotype of *P. fruitensis.* A, lingual view (tooth mirrored for better comparison) and B, occlusal view of M3; C, buccal view and D, occlusal view of right m3. The dark red area represents the part of the anteriorly inclined wear facet on the mesial side of cusp a, that still has enamel preserved, though the rest of the facet has dentine exposed. Scale bar equals 1 mm.

Supplementary Figure 3

Collision detection on the right m3 with M3 and M4 during the masticatory cycle of *P*. *fruitaensis*. Different stages of the power stroke. A, early; B, middle; C, late; in occlusal, lingual, buccal and mesial view. Due to the small difference in cusp height, all cusps come into contact at the beginning of the power stroke. With the passing of the crests, most of the cutting function takes place in the first quarter of the power stroke. Food at this stage either is cut off (e.g. meat) or fragmented (e.g. insects). Subsequently, the food is compressed and sheared in the individual valleys between the teeth while the lower molar moves upwards. The movement is primarily orthal with a slight distal component following the valleys in the upper molars. Roll during the power stroke is approximately 10° as seen from mesial. Arrows indicate mesial direction.

Supplementary Figure 5

Right maxilla and mandible of *P*. *fruitaensis* in A, medial view; B, anterior view; C, section from anterior view. The dentitions are interlocked but no roll was applied to the jaw (the ultimate premolars are facing each other vertically and the condyle is horizontal). The dotted line represents the plane for the section seen in C. The anterior region and parts of the maxilla were removed to highlight the molar positioning within the maxilla and lower ramus. The posterior upper molars are inclined lingually and the lower molars buccally to a lesser degree. This inclination reduces the roll required to keep the teeth in contact during the power stroke and provides a better cutting edge alignment with the crests of the lower and upper molars passing each in a more efficient angle.

Supplementary Figure 6

3D model of the right mandible of the holotype of *P*. *fruitaensis* in medial view. The anterior part of the dental canal and the masseteric foramen are highlighted. The masseteric foramen extends and narrows towards anterior until it projects ventrally and connects with the dental canal underneath the anterior root of m3. Other canals were not highlighted due to better visibility, most notably a branch that located underneath the masseteric foramen is also connected to the dental canal, but does not open in a foramen on the outer surface. The distal part extends towards posterior close to the condyle of the dental canal, but not highlighted due to damage.

Supplementary Figure 7

Interpretation of mandibular adductor muscle attachment in A, *P*. *fruitaensis*; B, *Trioracodon ferox* (NHMUK PV OR 47775); and C and D, *Triconodon mordax* (NHMUK PV OR 47764 and 47763) in medial and lateral view. Muscular insertions that are assigned tentatively are marked (?) and in some cases, an alternative position is mentioned. While the surface area is nonindicative, morphological borders where used to estimate the muscle insertion areas by comparison to extant mammals (Barghusen 1968; Turnbull 1970; Lautenschlager et al. 2017).
