# Supplementary figures and images for: Molar occlusion and jaw roll in early crown mammals
Source: Sci Rep. 2020 Dec 24;10:22378. doi: 10.1038/s41598-020-79159-4 (PMC7759581; doi:10.1038/s41598-020-79159-4)

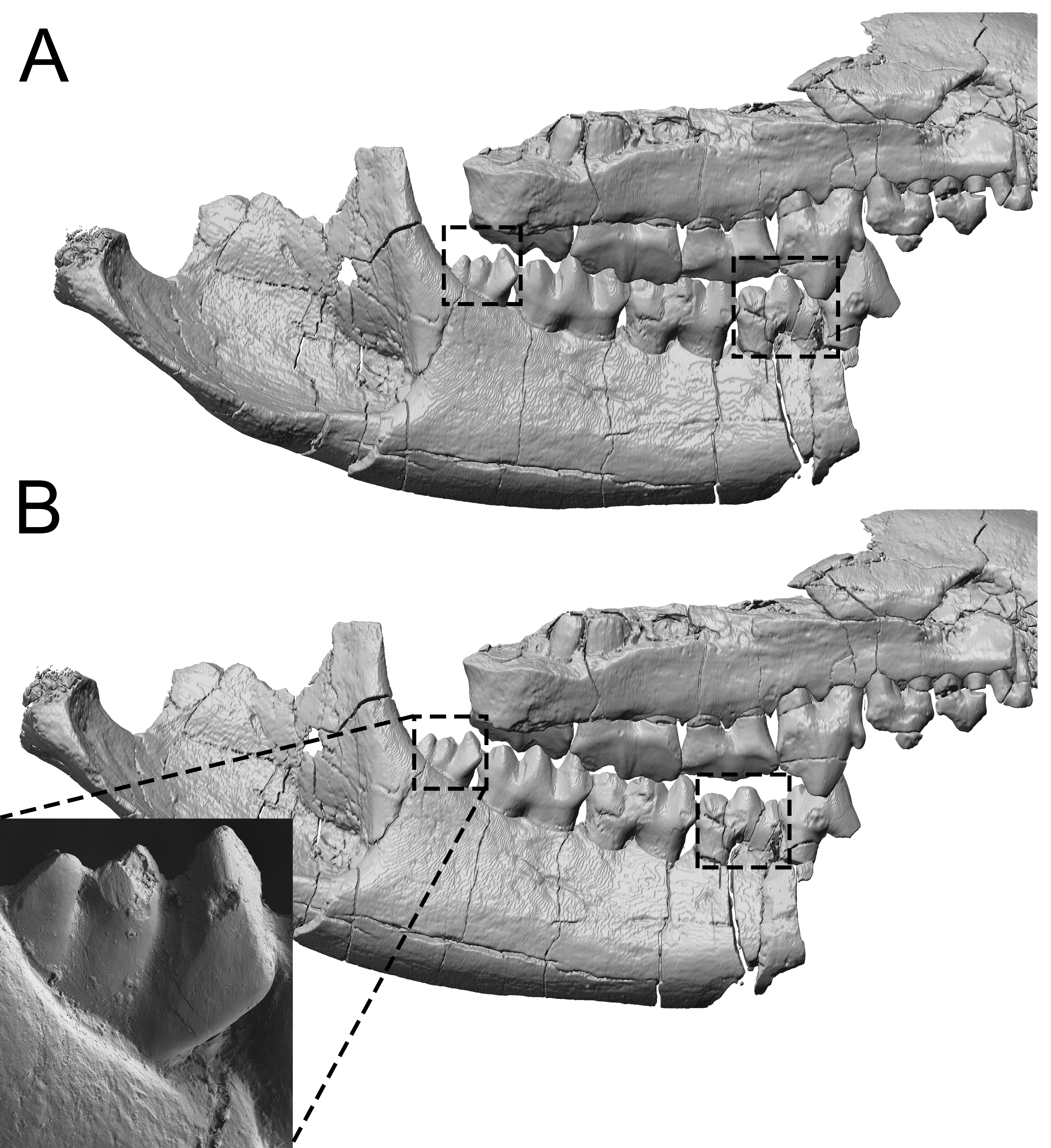

Supplement: Supplementary file 2 — Supplementary Information 2. [file 41598_2020_79159_MOESM2_ESM.jpg]

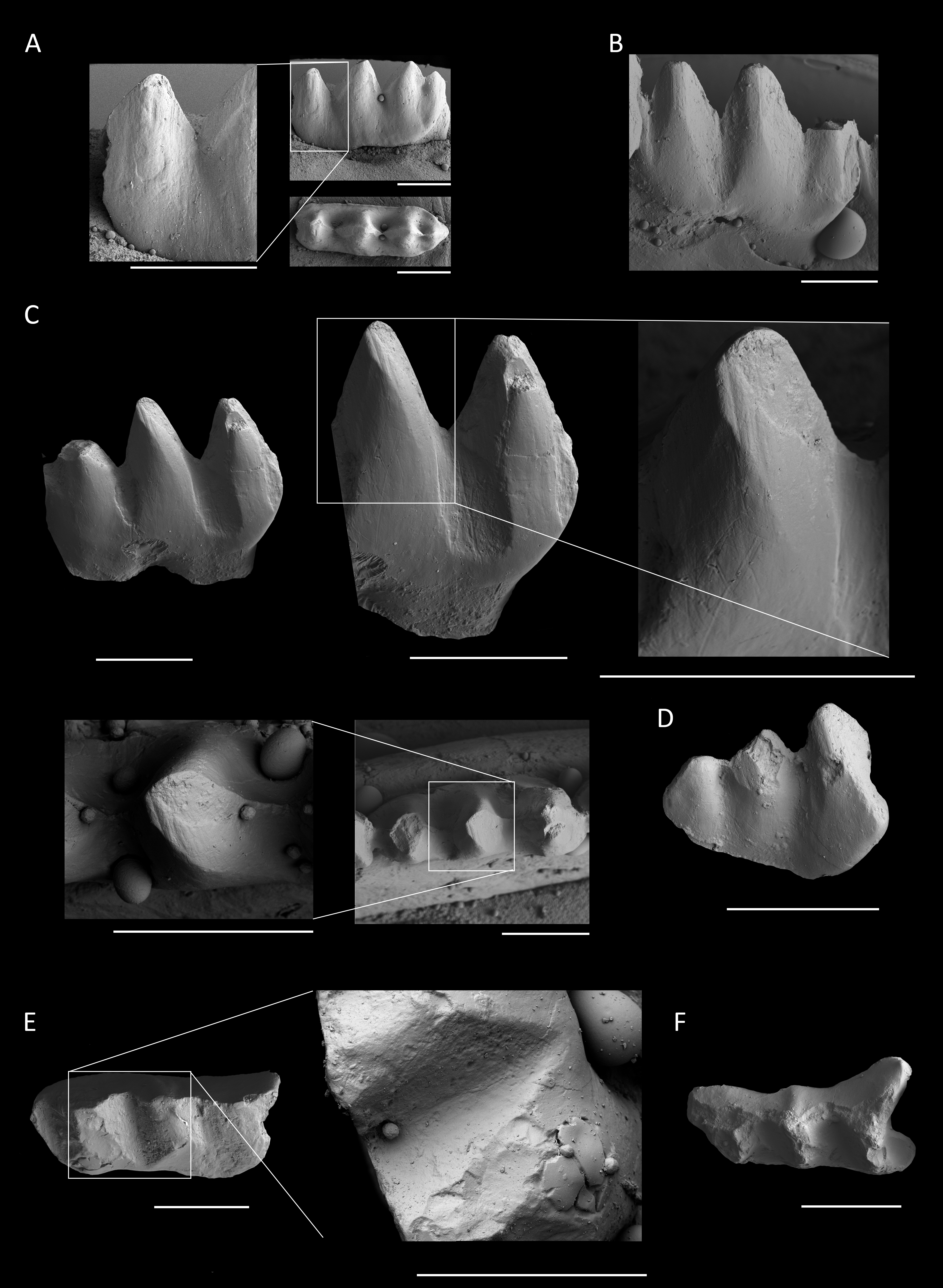

Supplement: Supplementary file 3 — Supplementary Information 3. [file 41598_2020_79159_MOESM3_ESM.jpg]

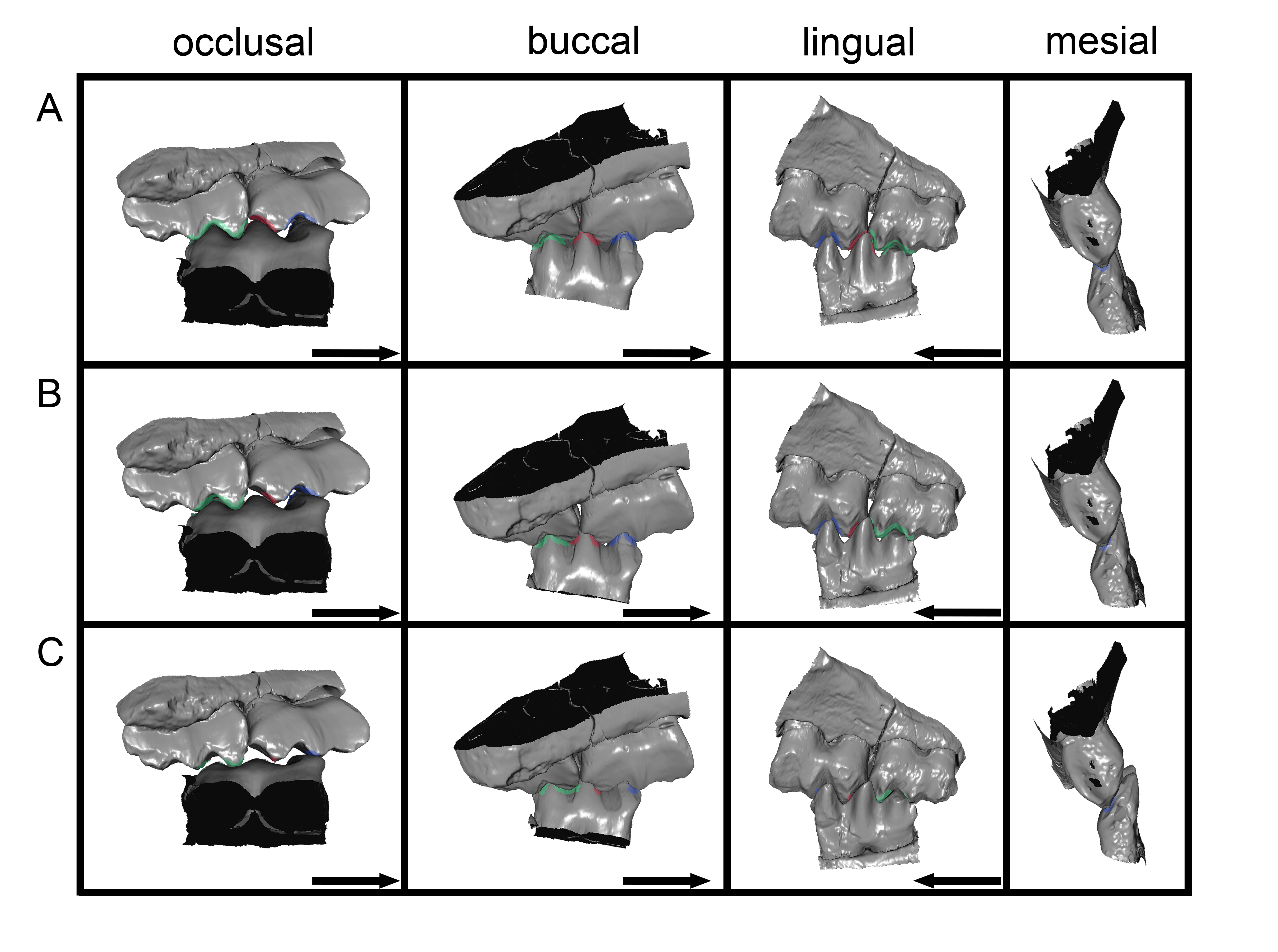

Supplement: Supplementary file 4 — Supplementary Information 4. [file 41598_2020_79159_MOESM4_ESM.jpg]

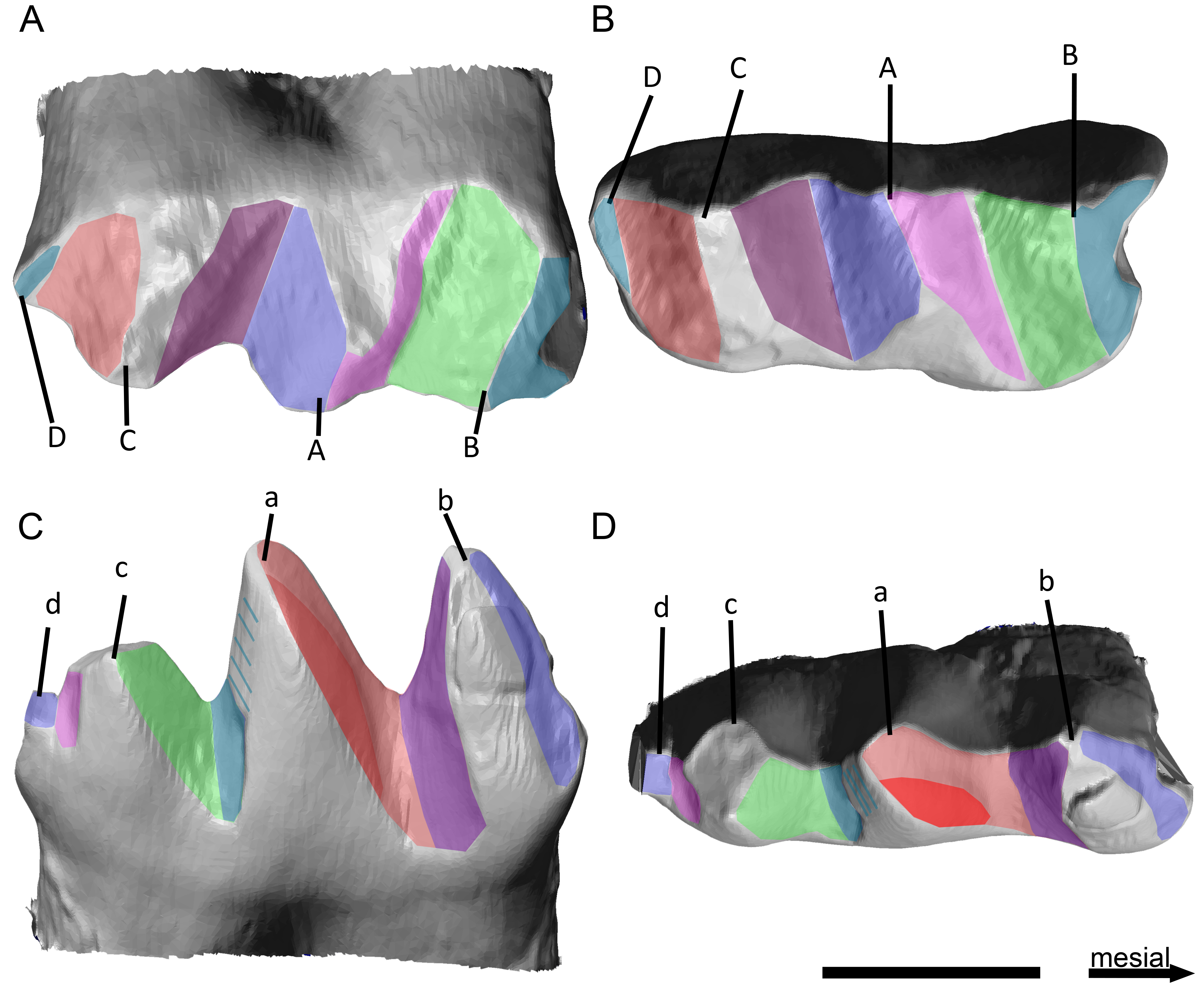

Supplement: Supplementary file 5 — Supplementary Information 5. [file 41598_2020_79159_MOESM5_ESM.jpg]

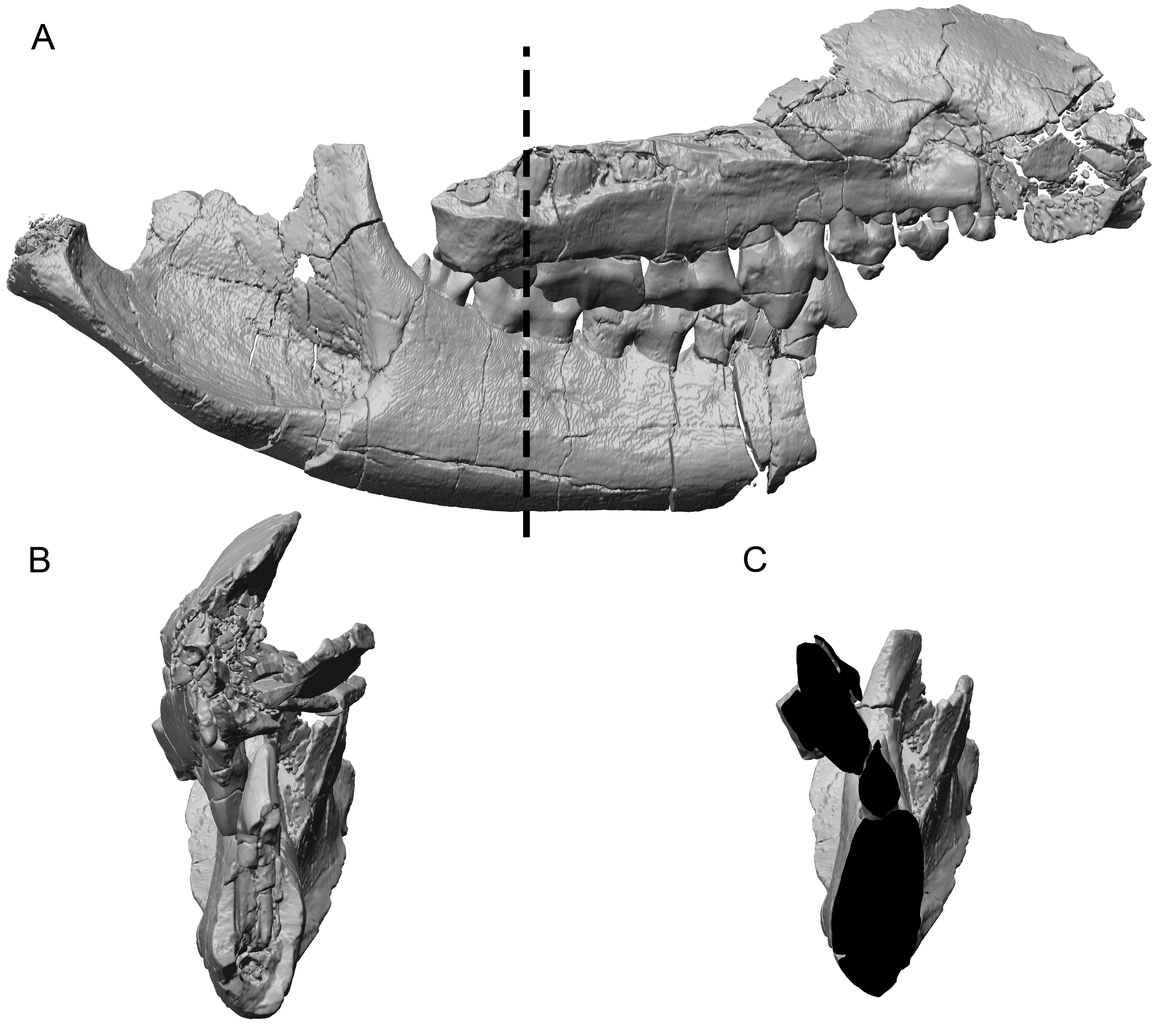

Supplement: Supplementary file 6 — Supplementary Information 6. [file 41598_2020_79159_MOESM6_ESM.jpg]

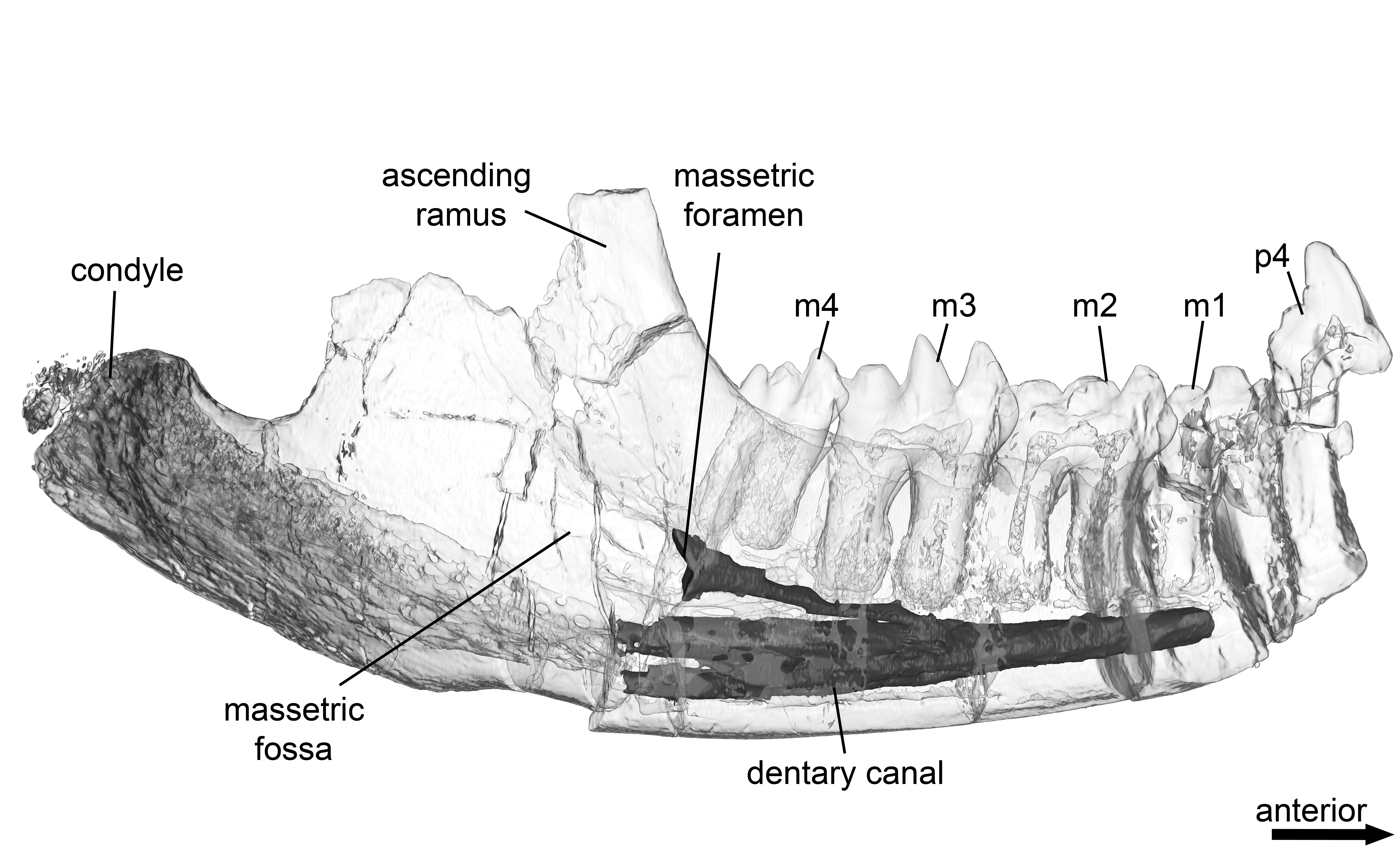

Supplement: Supplementary file 7 — Supplementary Information 7. [file 41598_2020_79159_MOESM7_ESM.jpg]

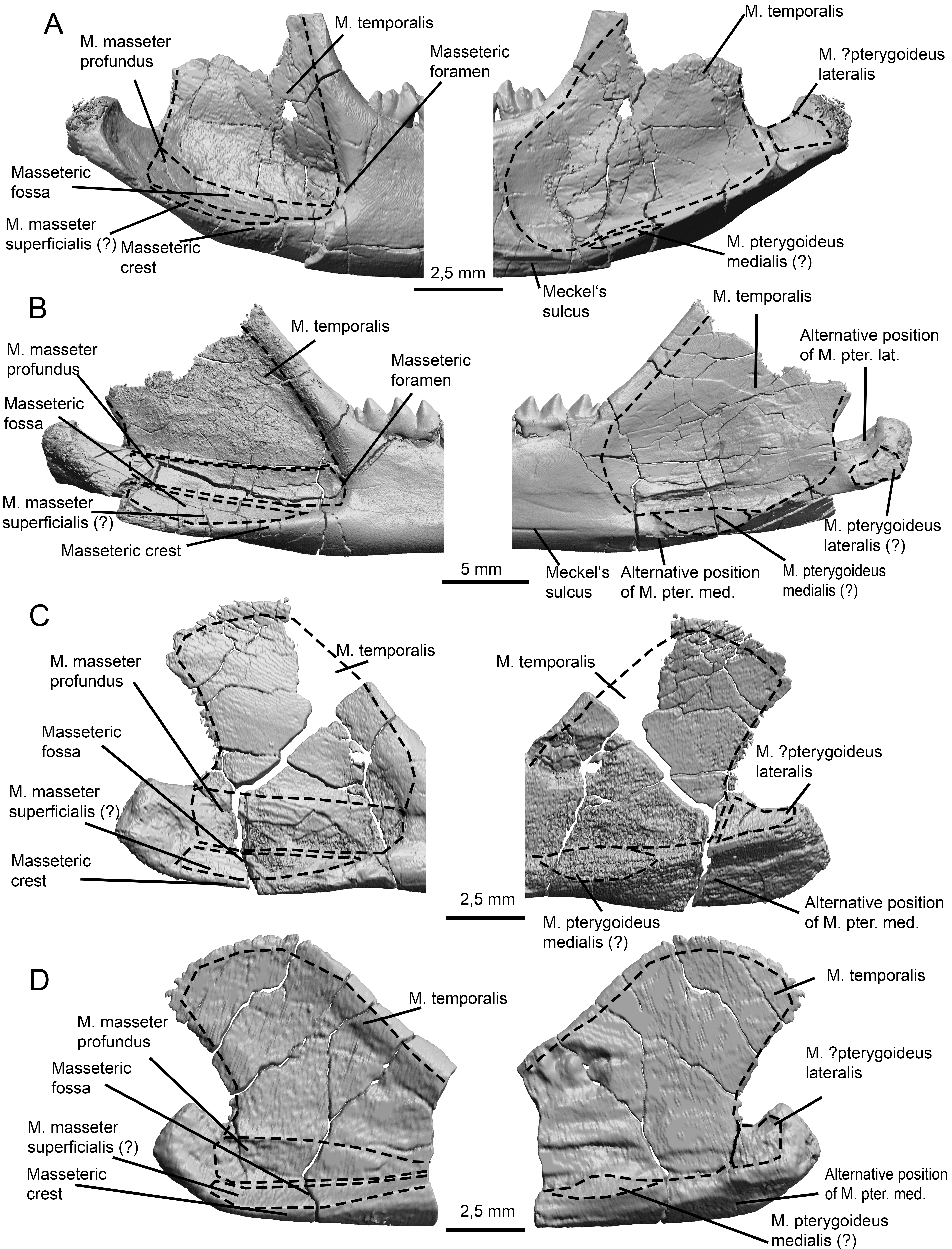

Supplement: Supplementary file 8 — Supplementary Information 8. [file 41598_2020_79159_MOESM8_ESM.jpg]
